# Axially‐Polarized Excitonic Series and Anisotropic van der Waals Stacked Heterojunction in a Quasi‐1D Layered Transition‐Metal Trichalcogenide

**DOI:** 10.1002/advs.202406781

**Published:** 2024-08-05

**Authors:** Adzilah Shahna Rosyadi, Ying‐Xuan Lin, Yu‐Hung Peng, Ching‐Hwa Ho

**Affiliations:** ^1^ Graduate Institute of Applied Science and Technology National Taiwan University of Science and Technology Taipei 106 Taiwan; ^2^ Taiwan Consortium of Emergent Crystalline Materials (TCECM) National Science and Technology Council Taipei 106 Taiwan

**Keywords:** 2D semiconductor, excitons, in‐plane anisotropy, optical property, polarized van der waals stacked device

## Abstract

Anisotropic optical 2D materials are crucial for achieving multiple‐quanta functions within quantum materials, which enables the fabrication of axially polarized electronic and optoelectronic devices. In this work, multiple excitonic emissions owning polarization‐sensitive orientations are clearly detected in a multilayered quasi‐1D ZrS_3_ nanoribbon with respect to the nanostripe edge. Four excitons denoted as A_S1_, A_S2_, A_S_, and A_2_ with E ⊥ *b* polarized direction and one prominent A_1_ exciton with E || *b* polarized emission are simultaneously detected in the polarized micro‐photoluminescence (µPL) measurement of 1.9–2.2 eV at 10 K. In contrast to light emission, polarized micro‐thermoreflectance (µTR) measurements are performed to identify the polarization dependence and verify the excitons in the multilayered ZrS_3_ nanoribbon from the perspective of light absorption. At 10 K, a prominent and broadened peak on the lower‐energy side, containing an indirect resonant emission (D_I_) observed by µPL and an indirect defect‐bound exciton peak (A_Ind_) observed by both µPL and µTR, is simultaneously detected, confirming the existence of a quasi‐direct band edge in ZrS_3_. A van der Waals stacked *p*‐GaSe/*n*‐ZrS_3_ heterojunction solar cell is fabricated, which demonstrates a maximum axially‐polarized conversion efficiency up to 0.412% as the E || *b* polarized light incident onto the device.

## Graphical abstract

Multiple excitonic‐series emissions possess dissimilar polarization‐sensitive orientations are clearly present in a multilayered quasi‐1D ZrS_3_ nanoribbon with respect to the nanostripe edge of *b* axis. The excitonic emissions with E || *b* and E ⊥ *b* polarizations are respectively identified. A *p*‐GaSe/*n*‐ZrS_3_ stacked‐junction solar cell is also fabricated to show a highest polarized efficiency along the ZrS_3_ nanoribbon's *b* axis.

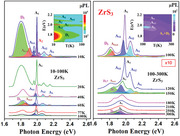

## 1 Introduction

2D materials with in‐plane anisotropy offer multiple quantum states through various mechanisms, such as manifold excitonic‐transition energies,^[^ ^1^ ^]^ orthogonal polarization directions (i.e., E parallel and E perpendicular to a specific axis),^[^ ^2^, ^3^, ^4^ ^]^ changes in the spin order of 2D layered magnets,^[^ ^5^ ^]^ and alterations in the ferroelectric state in 2D dielectric materials, etc.^[^ ^6^ ^]^ These materials can be considered as new quantum materials beyond the commonly studied superconductors. The rich quantum states of these materials can be utilized for high‐speed quantum computation and big data processing, distinct from traditional microprocessors that handle only digital states of “0” and “1” electrically.

Among the 2D materials, graphene has been the subject of numerous groundbreaking studies in integrated electronics (photonics) and optics technology. However, graphene's zero‐bandgap nature limits its applications in certain areas. Alternatively, monolayer and few‐layer 2D transition metal dichalcogenides (TMDCs) have gained significant attention due to their bandgap tunability and versatility in various fields. To date, MoS_2_, MoSe_2_, WS_2_, and WSe_2_ are the most frequently studied TMDCs due to their in‐plane and out‐of‐plane structural symmetries and diversed applications.^[^ ^7^, ^8^ ^]^ These TMDCs usually have bandgaps aligned only within the visible‐light absorption range, making them unsuitable for infrared optoelectronics.^[^ ^9^ ^]^ Additionally, larger‐bandgap TMDCs (>1.5 eV) often exhibit relatively lower carrier mobility (10–200 cm^2^/V‐s) compared to graphene.^[^ ^10^, ^11^, ^12^ ^]^ To bridge the gap between graphene (zero‐gap) and TMDCs (near‐infrared‐visible range), black phosphorus (BP) is a promising candidate for near‐ to mid‐infrared optoelectronic devices and exhibits in‐plane anisotropic behavior.^[^ ^9^ ^]^ BP's anisotropic nature drives the demand for novel materials with directional or axial dependencies for optical, electrical, and optoelectronic applications. Notably, ReS_2_ and ReSe_2_ TMDCs,^[^ ^13^ ^]^ as well as group III/IV‐VI compounds like GaTe^[^ ^14^ ^]^ and SnSe,^[^ ^15^ ^]^ are extensively studied for their highly anisotropic structures and optical and electrical asymmetric behaviors. Despite being less prominent, transition metal trichalcogenides (TMTCs) also show significant anisotropic behavior in their optical and electrical properties due to their quasi‐1D structures but have not yet been systematically studied.

ZrS_3_, belonging to the group‐IV TMTCs (TM = Ti, Zr, and Hf), has a quasi‐1D crystal structure with the space group *P*2_1_/*m*. Its strong in‐plane anisotropy makes it suitable for electronic and optoelectronic applications due to its high anisotropic ratios in conductivity and axial dichroism.^[^ ^16^ ^]^ ZrS_3_ exhibits a remarkable photoresponse across a wide range of wavelengths, from ultraviolet to near‐infrared light.^[^ ^16^, ^17^ ^]^ It also possesses high stability for operation in air.^[^ ^18^ ^]^ Unlike its TMDC counterpart, the ZrS_2‐x_Se_x_ series, which is unstable and prone to oxidation in the van der Waals plane,^[^ ^19^ ^]^ ZrS_3_ has a more stable crystal surface. ZrS_3_ is considered an indirect semiconductor, and its photoluminescence behavior is controversial and not well understood, as indicated by many theoretical band‐structure calculations previously.^[^ ^20^, ^21^, ^22^ ^]^ Few reports describe the origin of photoluminescence in ZrS_3_ due to its indirect gap environment. Pan et al. observed a broadened photoluminescence in ZrS_3_ at ≈1.8 eV,^[^ ^22^ ^]^ although the direct band edge of TMTC is near 2 eV. An earlier study by Ait‐Ouali et al. found that photoluminescence peaks at low temperature appear ≈1.96 – 2.08 eV and that the luminescence bands are sensitive to small temperature changes and quenched over time.^[^ ^23^ ^]^ The PL signal at ≈2 eV was observed in ZrS_3_ after heat treatment,^[^ ^24^ ^]^ and it was attributed to zirconium oxide related peak.^[^ ^24^, ^25^ ^]^ The light‐emission properties of ZrS_3_ remain unrevealed, and there is no clear consensus on the attribution and mechanism of the exciton‐series emissions in axially polarized multilayered ZrS_3_.

We demonstrate herein strong visible excitonic lines (denoted as A_S1_, A_S2_, A_S_, A_1_, and A_2_) emitted from multilayer (ML) ZrS_3_ nanoribbons between 1.9 and 2.3 eV using polarized micro‐photoluminescence (µPL) measurement at 10 K. Additionally, there is a broadened PL peak at 1.8 eV (denoted as D_I_) that may result from an indirect‐like layer‐by‐layer resonant emission at 10 K. The PL peaks A_1_ and D_I_ show predominant E || *b* polarized emission, while A_S1_, A_S2_, A_S_, and A_2_ features mainly emit E ⊥ *b* polarized light, as observed in angular‐dependent polarized µPL measurements at 10 K. The excitonic series transitions of ML‐ZrS_3_ with similar energy and polarization can also be detected and verified through experimental band‐structure measurements using polarized micro‐thermoreflectance (µTR) experiments at 10 K. The layered ZrS_3_ is proposed to possess an indirect‐gap nature in both monolayer and bulk forms,^[^ ^20^, ^21^, ^22^ ^]^ the observation of excitonic‐series emissions suggests that ML‐ZrS_3_ nanoribbons should be considered as a “quasi‐direct” semiconductor. This property differs from TMDCs like MoS_2_, MoSe_2_, WS_2_, and WSe_2_,^[^ ^26^ ^]^ which exhibit thickness‐dependent band structures from bulk to monolayer. The experimental band‐edge structure of ZrS_3_ shows some resemblance to ReS_2_ and ReSe_2_,^[^ ^27^, ^28^, ^29^ ^]^ which simultaneously present indirect and direct PL emissions in their bulk and ML forms.^[^ ^28^, ^29^ ^]^ According to previous experimental and theoretical reports on ReS_2_, the small *k*‐difference between the valence band maximum (VBM) and conduction band minimum (CBM) allows monolayer and bulk ReS_2_ to emit strong PL peaks at room temperature.^[^ ^30^, ^31^ ^]^ The polarized excitons (A_S1_, A_S2_, A_S_, A_1_, and A_2_) emitted from ML‐ZrS_3_ nanoribbons may originate from the Γ point with Γ‐X or Γ‐Y oriented polarizations. The theoretical CBM of ML‐ZrS_3_ is at Z point, indicating an indirect transition, i.e., a “quasi‐direct band.” The transition assignments of µPL and µTR are referred to density‐function‐theory (DFT) band structure calculations. Temperature‐dependent µPL, µTR, and micro‐transmittance measurements of ML‐ZrS_3_ at 10–300 K are implemented to evaluate the excitonic transition energies, indirect and quasi‐direct band edges, and excitonic binding energies of the TMTC. Time‐resolved photoluminescence (TRPL) with area‐mapping function is used to characterize ML‐ZrS_3_ in the energy region near the main exciton peak of A_1_ from 10 to 300 K. The PL decay lifetime is determined to be τ_1_ = 0.31 ± 0.02 ns at 300 K. The fast recombination time indicates the emission originates from the direct band edge of layered ZrS_3_. Based on the axial‐polarized and in‐plane anisotropic behaviors of ML‐ZrS_3_, a stacked multilayer *p*‐GaSe Cd 1%/*n*‐ZrS_3_ heterojunction solar cell (SC) was fabricated, showing different photoelectric conversion efficiencies with linearly‐polarized light parallel and perpendicular to the ML‐ZrS_3_ nanoribbon's *b*‐axis.

## 2 Results and Discussion

**Figure 1:**
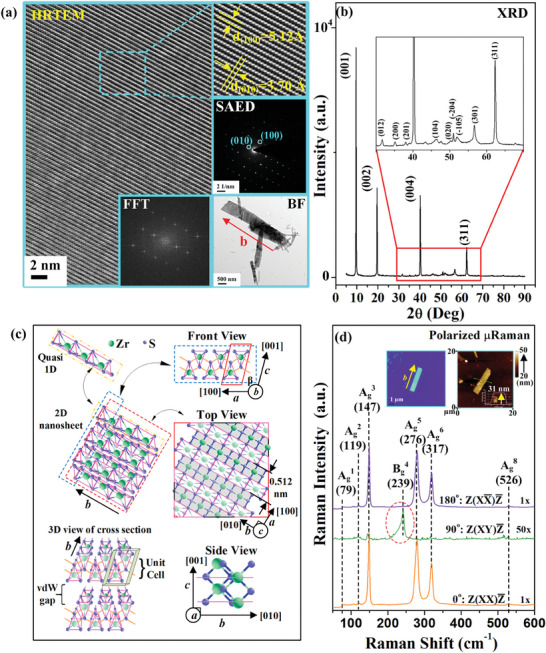
a) High‐resolution transmission electron microscope (HRTEM) image, selection‐area electron diffraction (SAED) pattern, bright field (BF) mode of the ZrS_3_ nanoribbons, and also fast fourier transform (FFT) result of the multilayered ZrS_3_ nanoflakes. b) The powdered X‐ray diffraction pattern of ML‐ZrS_3_ in the 2θ angle range of 5–90°. The enlarged portion is the peak pattern at the 35–70° range. c) The atomic arrangement seen from the side views of *a* and *b* axis, and top view in ML‐ZrS_3_. The 3D atomic schemes that responsible for the quasi‐1D chain structure (left‐top), 2D nanosheet (left‐middle), and 3D cross section view with vdW gap (left‐bottom) are included for illustration. d) The polarized micro‐Raman (µRaman) spectra with the polarized angle of 0°, 90°, and 180° with respect to the ZrS_3_ nanoribbon's *b* axis. The OM image and AFM result of the nanoribbon are also displayed in the inset.

**Figure** **1**a shows the high‐resolution‐transmission‐electron‐microscope (HRTEM) image derived from an as‐grown few‐layer ZrS_3_ nanoribbon flake (with crystal edge along *b* axis) shown in the inset. The result clearly shows stripe fringes along the crystal's *b* axis and the magnification image from a square region (see the right‐upper part) displays the lattice spacing values are 3.7 Å for *d*_(010)_ and 5.12 Å for *d*_(100)_, respectively. The selected‐area‐electron‐diffraction (SAED) pattern reveals dotted pattern with the marked index planes of (010) and (100) match well with the HRTEM lattice spacing and reported data.^[^ ^32^ ^]^ The SAED result also agrees with the fast‐Fourier‐transform (FFT) pattern derived from the HRTEM result in Figure 1a. The HRTEM and SAED results confirmed highly crystalline quality of the ML‐ZrS_3_ nanoribbon available for further optical and electrical characterizations. Figure 1b presents the powdered X‐ray diffraction (XRD) pattern of ZrS_3_ crystal with the 2θ angle ranges from 5 to 90°. The preferred orientation of the layer structure is presented as the *c*‐plane indices series of (001), (002), and (004) peaks. The magnification section of 2θ = 30°−70° with marked indices are also displayed in the inset square of Figure 1b for illustration. The XRD pattern matches well with the standard diffraction data (JCPDS #30‐1498) with a monoclinic symmetry (*P*2_1_/*m*) and the lattice constants are estimated to be *a* = 5.13 Å, *b* = 3.72 Å, *c* = 8.93 Å, and β = 97.48°, respectively. The values of in‐plane lattice constant *a* and *b* are in agreement with the HRTEM result shown in Figure 1a.

Figure 1c depicts the atomic schemes of *b*‐side view, top view, *a*‐side view, and 3D cross section view, etc. of ZrS_3_, where the green circle illustrates a Zr atom and the blue ball represents a sulfur ion. From the *b*‐side view, the fundamental unit of ZrS_3_ is consisted of one‐pair upside down ZrS_6_ trigonal prisms in the enclosed red area in Figure 1c. Each ZrS_6_ trigonal prism provides four triangular faces and the individual upside‐down pair interconnects with each other to extend along *b* axis for building pseudo‐1D chain‐like structure. The *a*‐side view of the lower‐right part in Figure 1c depicts a chain‐like structure to extend along [010] direction. The chain‐like structure can be regarded as a Quasi‐1D arrangement as shown in the 3D view in the left side of the *b*‐side view. The inter‐chain bond between the Zr and S atoms (along *a* direction) is weakly bonded. Therefore, the quasi‐1D feature is mainly responsible for the in‐plane anisotropy along and perpendicular to the *b*‐axis direction (i.e., force or electric field || and ⊥ to the *b* axis). For the atomic arrangement of the top‐view plane (also *c*‐plane) of ZrS_3_, it is similar to the HRTEM image in Figure 1a which possesses the spacing of chain about *a* = 5.12 Å. Figure 1c also presents a 3D cross section view of a two‐layer ZrS_3_ where the monoclinic unit cell and van der Waals gap are present.

To see the axial‐dependent structural anisotropy, polarized micro‐Raman (µRaman) measurement of a ML‐ZrS_3_ nanoribbon (exfoliated on SiO_2_/Si substrate) was carried out and the result was shown in Figure 1d with Z(XX) $\bar{Z}$ (θ = 0°, E || *b*), Z(XY) $\bar{Z}$ (θ = 90°, E ⊥ *b*), and Z(X $\bar{X}$) $\bar{Z}$ (θ = 180°, E || *b*) polarized configurations. Where E represents the optical electric‐field direction. The insets of Figure 1d show the atomic‐force‐microscope (AFM) image of the nanoribbon sample, which is in accordance with the optical‐microscope (OM) picture with a layer thickness of ≈31 nm, corresponding to 35 layers of ZrS_3_ (i.e., the monolayer thickness is ≈0.89 nm).^[^ ^32^ ^]^ There are at least 6 active out‐of‐plane modes of A_g_^1^ (79 cm^−1^), A_g_^2^ (119 cm^−1^), A_g_^3^ (147 cm^−1^), A_g_^5^ (276 cm^−1^), A_g_^6^ (317 cm^−1^), and A_g_^8^ (526 cm^−1^) with the maximum intensity appeared at the E || *b* (θ = 0° and 180°) polarized direction but they diminish significantly at the E ⊥ *b* (θ = 90°) polarization in Figure 1d.^[^ ^33^ ^]^ Specially, one of the in‐plane vibration mode B_g_^4^ (239 cm^−1^) reaches its maximum intensity at the E ⊥ *b* (θ = 90°) polarization and which disappears at the E || *b* polarization, in orthogonal to those of the A_g_ related modes in Figure 1d. The full angle‐dependent polarized µRaman spectra for each of the Raman modes from θ = 0° to 180° are demonstrated in Figure S1a (Supporting Information), and their atomic movements of Raman mode are also included in Figure S1b–h (Supporting Information) for comparison. The polar plot of each vibration mode is analyzed using angular‐dependent dichroic relation as:

(1) $$I\;\left(\theta \right)=I_{o}+I_{p}\times \mathrm{co}s^{2}\left(\theta -\theta_{m}\right)$$

where *I_o_* is the offset of the Raman intensity, *I_p_* the amplitude, and *θ*_m_ is the angle deviation from the *b* axis. The polar plots of fitting result of **Equation (1)** together with the attribution of each Raman mode of ZrS_3_ are listed in Table S1 (Supporting Information). The A_g_ related modes and the B_g_^4^ (239 cm^−1^) peak exhibit mutual orthogonal in polarized angle (*θ_m_*), which is typical for the monoclinic layer structure with quasi‐1D chains.

**Figure 2:**
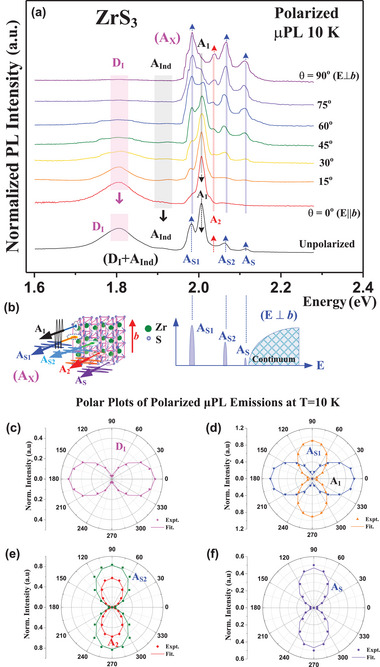
a) Angle‐dependent µPL spectra of the excitonic peaks in a ML‐ZrS_3_ nanoribbon from θ = 0° (E || *b*) to θ = 90° (E ⊥ *b*) at 10 K. b) The representative scheme of polarized emissions of the observed excitons (A_X_) is displayed in the left part. The right part depicts the indication of one detected excitonic series denoted as A_S1_, A_S2_, A_S_, and continuum band in the ZrS_3_ nanoribbon with *a*‐polarized behavior. The polar plots and angular‐dependent polarized‐emission analysis of c) D_I_, d) A_S1_, and A_1_, e) A_S2_ and A_2_, and f) A_S_ observed from the polarized µPL spectra at 10 K.

Owing to the reduced dimensionality of 2D layered materials, the screen effect to a many‐body system (like excitons and trions, etc.) is weakened for rendering a larger binding energy of excitons.^[^ ^34^ ^]^ Especially if the 2D materials have a lower structural symmetry as monoclinic or triclinic layer structure, plenty number of excitonic transitions, e.g. excitons in ReS_2_ and ReSe_2_,^[^ ^35^ ^]^ are present in the layers. **Figure** **2**a shows the unpolarized (bottom) and angle‐dependent polarized µPL spectra of θ = 0 (E || *b*), 15, 30, 45, 60, 75, and 90° (E ⊥ *b*) of a multilayered ZrS_3_ nanoribbon as the sample picture and AFM image shown in the inset of Figure 1d at 10 K. The polarized and unpolarized µPL spectra in Figure 2a show one broadened peak at D_I_ ≈ 1.805 eV, a smaller peak of A_Ind_ ≈ 1.9 eV, and the other group of sharp excitonic series (marked as A_X_) including the peak features of A_S1_ ≈ 1.984, A_1_ ≈ 2.004, A_2_ ≈ 2.035, A_S2_ ≈ 2.062, and A_S_ ≈ 2.111 eV with energy sequence present at 10 K. Observing in detailing in the PL peaks of the A_S1_, A_S2_ and A_S_ series, the three exciton peaks show an intensity‐decreased sequence from A_S1_ (highest), A_S2_ (middle) to A_S_ (minimum) and they show the same polarization dependence with the highest intensity at θ = 90° (E ⊥ *b*) and the minimum strength at θ = 0° (E || *b*) polarized spectrum as shown in the blue‐arrow directions for increasing amplitudes. These excitons are referred to a modified Rydberg series (exciton) in the ML‐ZrS_3_ with an E ⊥ *b* (or E || *a*) axially‐polarized transition along the Γ‐X direction close to the Γ point. The representative scheme of the modified Rydberg exciton series (A_S1_, A_S2_ and A_S_) with energy and intensity order (as observed from Figure 2a) is shown in the below plot in Figure 2b. The “continuum” band depicts the end‐band state of this Rydberg series. The energy separations between the A_S1_, A_S2_, A_S_ and continuum band can be further analyzed using modified Rydberg series formula, E_n_ = E_g_‐R_yd_^*^/(n+γ)^2^,^[^ ^36^, ^37^ ^]^ where E_n_ is the energy of the n_th_ peak (*n* = 1, 2, 3, …), E_g_ is the threshold energy of the continuum band, and R_yd_^*^ is the effective Rydberg constant (i.e., binding energy of the *n* = 1 level). The value of γ is related to the dimensionality (α) of the material expressed as γ = (α−3)/2.^[^ ^36^ ^]^ For the 3D case, i.e., α = 3 and γ = 0, the Rydberg series formula returns to Hydrogen like and the electron wave‐function operation can be only radius (r) dependent, ψ(r) = R(r). For the dimensionality lower than 3D (1<α<3), the value of γ is negative. The angular momentum will start to incorporate into the states and the electron wave function will become radius (r) and angle (θ) dependent, ψ(r,θ) = R(r)Θ(θ).^[^ ^36^ ^]^ According to the fitting analysis of the A_S_ series in Figure 2a using Rydberg series formula, the physical parameters can be obtained to be E_g_ = 2.13 ± 0.02 eV, R_yd_^*^ = 0.14 ± 0.01 eV, and γ = ‐(0.03 ± 0.02), respectively, at 10 K. The negative value of γ means the dimensionality of the ML‐ZrS_3_ is lower than 3. For the A_1_ feature, the PL peak intensity is the highest in the unpolarized spectrum and it shows an E || *b* polarized emission as the peak strength varied as the black‐arrow line shown in Figure 2a. The A_1_ can be regarded as the main band‐edge exciton coming from the direct gap along the Γ‐Y direction near the Γ point of the band structure in ML‐ZrS_3_. The transition amplitude of the A_2_ emission is small (red‐arrow line) and which is along the largely *a*‐polarized (θ = 90°) direction in Figure 2a, similar to the A_S_ series excitons. It may come from the VBM consisted of multi‐valley Δ degeneracy (Δ_1_, Δ_2_, etc.) along the Γ‐X direction near the Γ point in the quasi‐1D ML‐ZrS_3_ band. The origins of the A_1_, A_2_, and A_S_ series transitions will be evaluated and discussed by theoretical band‐structure calculation and experimental critical‐points transitions observed by µTR experiment later.

For the broadened PL feature D_I_ of ML‐ZrS_3_ in Figure 2a, the peak may correlate with the multilayered indirect‐like resonant emission in a quasi‐direct band structure of ZrS_3_.^[^ ^28^ ^]^ The smaller peak of A_Ind_ ≈ 1.9 eV besides D_I_ (≈1.805 eV) is an indirect defect‐bound exciton of donor caused by sulfur vacancy (V_S_) below the CBM of ML‐ZrS_3_. The existence of sulfur deficiency to cause V_S_‐bound excitonic emission can be verified by the result of energy dispersive X‐ray spectroscopy of an as‐grown ZrS_3_ crystal (i.e., stoichiometric ratio Zr: S = 1: 2.92) shown in Figure S2a (Supporting Information). The X‐ray photoelectron spectroscopy (XPS) results of Zr 3d and S 2p states of ZrS_3_ are shown in Figure S2b,c (Supporting Information). Essentially, the Zr 3d (3d_3/2_ and 3d_5/2_) states shift to higher energy, while the S 2p (S 2p_1/2_ and S 2p_3/2_) orbitals shift to lower energy from the pure elements by forming a ZrS_3_ compound, as shown in Figure S2b,c (Supporting Information). In ZrS_3_, the sulfur 2p states include S 2p states [i.e., S^2−^ 2p_3/2_ (160.2 eV) and S^2−^ 2p_1/2_ (161.2 eV) peaks] and S_2_ 2p states [i.e., S_2_^2−^ 2p_3/2_ (161.2 eV) and S_2_^2−^ 2p_1/2_ (162.35 eV)].^[^ ^38^ ^]^ The XPS peak intensities of S 2p and S_2_ 2p in ZrS_3_ need to be comparable in an ideal sulfide compound of ZrS_3_. Figure S2c (Supporting Information) shows that the intensities of the S 2p states [S^2^⁻ 2p_3/2_ and S^2^⁻ 2p_1/2_] are lower than those of the corresponding S_2_ 2p states [S_2_^2−^ 2p_3/2_ and S_2_^2−^ 2p_1/2_], indicating that ZrS_3_ may exhibit only S vacancies and not S_2_ vacancies. The presence of S vacancies also results in the formation of a little ZrO_2_, as evidenced by a small shoulder peak at 183.6 eV in Figure S2b (Supporting Information).^[^ ^38^ ^]^ The O 1s peak in Figure S2d (Supporting Information) also proves a little bit oxidation in the crystal. The PL features D_I_ and A_Ind_ simultaneously reveal *b*‐polarized behavior at θ_m_ = 0° shown in the pink‐arrow line and black‐arrow line in Figure 2a. They are correlated with the optical contribution from indirect gap with an indirect‐like resonant emission (D_I_) and an indirect donor‐bound exciton (A_Ind_). The *b*‐polarized optical behavior of D_I_ and A_Ind_ is evident by the polarized transmittance and absorption spectra in Figure S3 (Supporting Information), where they showed a *b*‐polarized indirect band edge of ZrS_3_. The polar plots of angular dependence of the polarized µPL emissions in Figure 2a can be analyzed using the dichroic relation of Equation (1) and which are respectively displayed in Figure 2c–f for the features of D_I_, A_S1_, A_1_, A_2_, A_S2_, and A_S_. Essentially, the indirect gap (D_I_) and direct gap (A_1_) show E || *b* polarized behavior while the As series (A_S1_, A_S2_, and A_S_) and A_2_ exciton display an *a*‐polarized character to make multidisciplinary optical‐quanta states observed in ML‐ZrS_3_. A representative scheme of polarized light emissions from a ML‐ZrS_3_ is depicted in the left part of Figure 2b, the Rydberg series (A_S1_, A_S2_, and A_S_) and the main A_1_ exciton show mutual orthogonal of the polarization state. It is caused by the in‐plane anisotropy arisen from the Quasi‐1D chain‐like structure of the ZrS_3_ nanoribbon.

**Figure 3:**
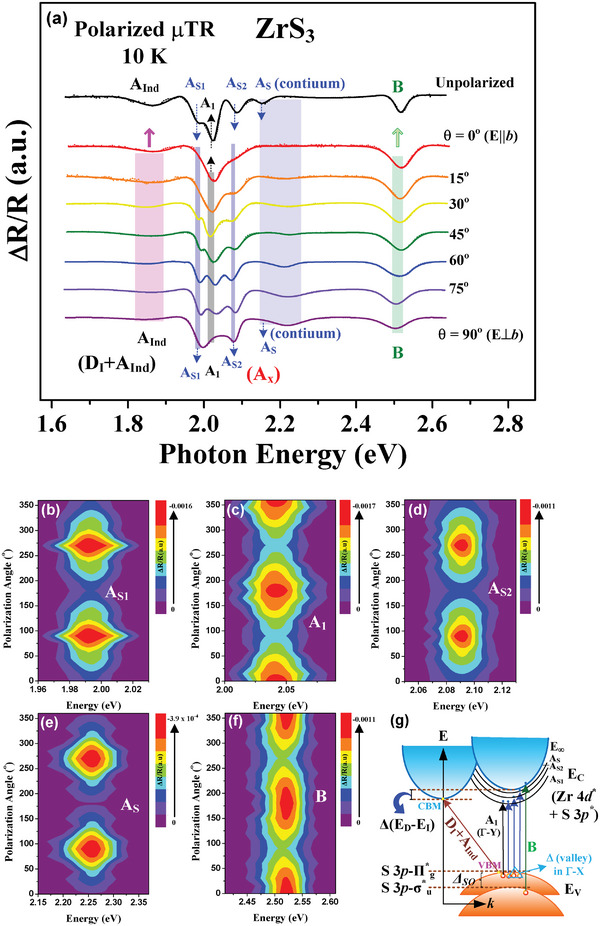
a) Angle‐dependent polarized µTR spectra measured from θ = 0° (E || *b*) to θ = 90° (E ⊥ *b*) for the band‐edge transitions in ML‐ZrS_3_ nanoribbon at 10 K. The transitions of A_Ind_, A_S1_, A_1_, A_S2_, A_S_, and B features (with different polarization dependence) are detected based on the optical‐absorption mechanism. The 2D contour plots of polarization dependence of the b) A_S1_, c) A_1_, d) A_S2_, e) A_S_, and f) B transitions are shown below. g) The band‐edge scheme of ML‐ZrS_3_ nanoribbon constructed by the experimental µPL and µTR measurements as well as theoretical band‐structure calculations in Figure S5 (Supporting Information).

**Figure 4:**
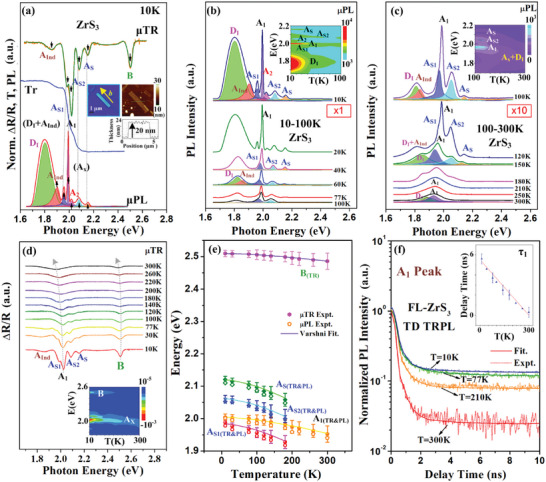
a) The comparison of unpolarized µTR, micro‐transmittance (Tr) and µPL spectra for the band‐edge transitions of ML‐ZrS_3_ at 10 K. The insets show the OM image and AFM result of a new sample of ZrS_3_ nanoribbon used for unpolarized µPL measurement. The temperature‐dependent µPL spectra of b) 10–100 K and c) 100–300 K (with 10× magnification) in ML‐ZrS_3_ together with the peaks fitting analysis of the excitons and transitions. The related 2D contour‐plot spectra are also shown in their insets for contrast. d) Temperature‐dependent µTR spectra (including 2D contour‐plot spectra) of ML‐ZrS_3_ between 10 and 300 K. e) The analysis of temperature‐dependent transition energies of ZrS_3_ obtained by µPL and µTR. The solid lines are those of the Varshni fits. f) Temperature‐dependent TRPL results (focused at the A_1_ peak) and the analysis of band‐edge recombination lifetime of ZrS_3_.

In order to verify the origin of the observed excitonic‐series emissions in the A_X_ group and investigate the indirect related D_I_ and A_Ind_ peaks, power‐dependent PL measurement was implemented. Figure S4a (Supporting Information) depicted power‐dependent PL spectra of multilayer ZrS_3_ using a 375‐nm solid state laser with different laser power from 0.3 mW (1%) to 30 mW (100%) at 25 K near band edge. Four main PL emissions of D_I_, A_Ind_, A_S1_, and A_1_ are observed and their peak intensities versus laser powers are displayed in Figure S4b (Supporting Information). The power dependence of each PL feature (solid line) can be analyzed using a law of I ≈ L^k^ [I is the PL intensity and L is the laser excitation power].^[^ ^39^ ^]^ The obtained fitted values for the indirect related parts of D_I_ and A_Ind_ are k ≈ 1.07 and k ≈ 1.09. For the free‐exciton related emissions, the k values are k ≈ 1.18 and k ≈ 1.13 for A_1_ and A_S1_. The k values of free‐exciton emissions are slightly larger than those of the indirect‐related emissions in ZrS_3_. For a non‐excitonic and defect‐related emission the k value is usually less than one (e.g., 0.6–0.7 in CdTe/GaAs).^[^ ^39^ ^]^ To further identify the µPL emission features in Figure 2a, light absorption (reflection) by optical transitions via polarized µTR experiment was also carried out. Modulated TR measurement of semiconductor is effective for characterization of excitons, critical‐point and inter‐band transitions in the semiconductor's band structure.^[^ ^40^, ^41^, ^42^ ^]^ It is a physically derivative method for measuring the reflectance spectrum of semiconductor dielectric function by directly applying heat modulation to the crystal lattice of the sample periodically. **Figure** **3**a shows the polarized and unpolarized µTR spectra of the ML‐ZrS_3_ nanoribbon sample (as that in the µPL measurement) from 1.65 to 2.65 eV at 10 K. There are a lot of transition features denoted as A_Ind_, A_S1_, A_1_, A_S2_, A_S_, and B features can be detected in the unpolarized and angle‐dependent polarized µTR spectra from θ = 0° (E || *b*) to θ = 90° (E ⊥ *b*) as shown in Figure 3a. The dotted lines in Figure 3a are the experimental µTR spectra and the solid curves are those of the first derivative Lorentzian line‐shape fits of the experimental data using the expression as ΔR/R = *Re*{Σ_i = 1 to n_ I_i_^ex^/[*exp*(−*j*ϕ_i_^ex^)⋅(E‐E_i_^ex^+*j*Γ_i_^ex^)^2^]}.^[^ ^42^ ^]^ Where i is the respective transition, I_i_^ex^ and ϕ_i_^ex^ are amplitude and phase of the line shape, and E_i_^ex^ and Γ_i_^ex^ are the transition energy and broadening parameter of each band‐edge excitonic transition. The obtained transition energies from the line‐shape fits of the polarized and unpolarized µTR features at 10 K are A_Ind_ = 1.90 ± 0.03, A_S1_ = 1.986 ± 0.006, A_1_ = 2.008 ± 0.006, A_S2_ = 2.068 ± 0.008, A_S_ = 2.128 ± 0.062, and B = 2.51 ± 0.01 eV, respectively. The energies of the A_S1_, A_S2_, and A_S_ series excitons and that of the A_1_ transition measured by µTR are matched with those obtained in the µPL spectra of ML‐ZrS_3_ in Figure 2a. As shown in Figure 3a, the D_I_ feature of µPL is not clearly detected in the µTR measurement due to its much‐broadened and indirect character (see Figure S4a, Supporting Information) so that it cannot be sharply resolved in the derivative‐like TR spectrum. Thus, only the A_Ind_ transition can be detected in the ZrS_3_ nanoribbon at low temperature. Besides, the A_2_ transition of µPL in Figure 2a is weakened and not detected in the µTR spectra by thermal modulation in Figure 3a. It is maybe owing to the A_2_ exciton is very sensitive to the temperature change and it can be only detected at T ≤ 20 K using temperature‐dependent µPL as evident in **Figure** **4**b. For the A_S1_, A_S2_, A_S_ excitons and continuum band observed in µTR measurement at 10 K (see Figure 3a), the unpolarized µTR spectrum shows clearly the intensity order of A_S1_, A_S2_, and A_S_ as the µPL peaks shown in Figure 2a,b. However, for the polarized µTR spectra at θ = 15° to 90°, the transition amplitude of the latest As transition is weakened and reveals a broadened feature. This result may be due to the combination of A_S_ and the continuum band under the weakened polarized light of the incident probe beam in the polarized µTR condition, as displayed in Figure 3a.

The angular dependences of polarized intensities of µTR obtained for the A_S1_, A_1_, A_S2_, A_S_, and B transitions are analyzed and depicted in Figure 3b–f as the 2D contour plots. The 2D contour plot of the A_S_ series excitons (A_S1_, A_S2_ and A_S_) also demonstrates *a*‐polarized (E ⊥ *b*) behavior similar to the polarized µPL result in Figure 2d–f. The main band‐edge exciton A_1_ is also present *b*‐polarized behavior in Figure 3c, agrees with the polar plot of polarized µPL in Figure 2d. The A_Ind_ transition in Figure 3a is related to an indirect‐like bound exciton in this “quasi‐direct” layered material with a largely *b*‐polarized orientation at low temperature. There is also an extra feature B at ≈2.51 eV can be detected in the µTR spectra at 10 K in Figure 3a. This B exciton feature may come from the valence‐band splitting to conduction band valley of ZrS_3_, similar to the B exciton of the other TMDs like WS_2_ and MoS_2_, which originates from the split VB to CBM.^[^ ^43^ ^]^ From Figure 3a, the B exciton in ZrS_3_ cannot be fully forbidden in the polarized µTR measurement. It shows a majorly *b*‐polarized behavior (at θ ≈ 0° and 180°) as displayed in Figure 3f.

In order to support the assignment of transition origins of the band‐edge excitons, theoretical band calculations of the ML‐ZrS_3_ were implemented. Figure S5 (Supporting Information) shows the band‐structure calculations of a bulk ZrS_3_ using density function theory (DFT) method with some of the lattice parameters coming from the XRD measurement. The result proposes the multilayered ZrS_3_ is an indirect semiconductor with the valence‐band maximum (VBM) consisted of multi‐valley degeneracy points between the Γ and X positions (e.g., Δ_1_ and Δ_2_, etc.) whereas the conduction‐band minimum (CBM) is located at the Z point. The observation of multi‐valley degeneracy in the uppermost valence band of ZrS_3_ has also been reported in previous theoretical calculations,^[^ ^44^, ^45^ ^]^ occurring between the Γ and X points. The valence band maximum (VBM) of the quasi‐1D ZrS_3_ primarily comprises the *p*_x_ and *p*_y_ orbitals of S atoms within the partial density of states (PDOS). These orbitals exhibit spin degeneracy around the high‐symmetry Γ point, attributed to the inclusion of spin‐orbit coupling (SOC), resulting in Rashba‐like spin‐band splitting.^[^ ^46^ ^]^ As a result, the valence band undergoes significant spin‐band splitting, leading to the observation of multi‐Δ (Δ_1_, Δ_2_, Δ_3_, etc.) energy bands shifting during spin polarization. This phenomenon is evident in, for instance, the *a*‐polarized A_S_ series (A_S1_, A_S2_, and A_S_ excitons). The Rashba‐like physics in the band structure of condensed matter often arises from inversion breaking in a highly symmetric system, resembling hydrogen‐like behavior, and it will happen owing to the reduced dimensionality. The reduction of dimensionality could introduce angular momentum (L) into the Hamiltonian for wave function operations.^[^ ^46^ ^]^ For a structural transition from a pure 2D to a quasi‐1D structure of ZrS_3_, the Rashba effect can result in oriented multi‐valley peaks at the valence band maximum (VBM), which may not align with the Γ point but instead of lying along the Γ‐X or Γ‐Y directions. This result causes the in‐plane anisotropic properties of the ML‐ZrS_3_.^[^ ^46^ ^]^

Referred to the band‐structure calculation of Figure S5 (Supporting Information), the D_I_ transition observed in µPL is assigned to be an indirect‐resonant like feature originated from VBM at Γ‐Y to the CBM at Z. The A_Ind_ feature is an indirect donor‐bound exciton (by sulfur vacancy) which originates from VBM at Γ‐Y to the CBM at Z. For a clear illustration between the experimental and theoretical results of ML‐ZrS_3_, a representative scheme is thus depicted in Figure 3g for mutual comparison. The occurrence and observation of the excitonic series emissions of A_S1_, A_1_, A_2_, A_S2_, and A_S_ (A_X_) may come from the so‐call “quasi‐direct” or “direct‐like” excitonic transitions within the band structure of ML‐ZrS_3_ in Figure S5 (Supporting Information). The Rydberg like series of A_S1_, A_S2_, and A_S_ (as well as the other A_2_) with majorly *a*‐polarized behavior may originate from multi‐valley degeneracy Δ (VBM) → CBM along the Γ – X direction. The main band‐edge exciton A_1_ with a *b*‐polarized character is from VBM to CBM along the Γ to Y direction and that of B transition observed by µTR is from the spin‐orbital splitting (*Δ_SO_*) in ML‐ZrS_3_ with a slightly *b*‐polarized behavior. The experimental value of (VBM‐*Δ_SO_*) is ≈0.495 eV observed from µTR measurement in Figure 3a, which is in agreement with the calculated value of ≈0.5 eV in the band structure of Figure S5 (Supporting Information). The valence‐band state of ZrS_3_ is mainly contributed by the hybridization of S 3*p* orbitals with a small‐fraction contribution from the Zr 4*d* and 5*p* states. The highest occupied states of VBM of ZrS_3_ may split into two bands with the highest occupied states of antibonding S 3*p*‐Π^*^_g_ and the other lower states of S 3*p*‐σ^*^_u_^[^ ^47^ ^]^ with an energy separation of *Δ_SO_* ≈ 0.5 eV from µTR measurement. The CBM of the ML‐ZrS_3_ is mainly hybridized by the Zr 4*d* ^*^ and S 3*p* ^*^ antibonding states at Z point (see Figure 3g; Figure S5, Supporting Information). In general, the manifestation of photoluminescenece from the consisted excitonic series relies on the energy difference between the indirect and direct gap is relatively small in a “quasi‐direct” layered semiconductor.^[^ ^20^, ^21^ ^]^ As shown in the band structure of bulk ZrS_3_ in Figure S5 (Supporting Information), the energy difference of the two calculated CBM valleys (at Γ and Z) is very small [i.e., Δ(E_D_‐E_I_) ≈0.12 eV]. The value is close to the energy difference between the transitions of direct exciton A_1_ and indirect bound exciton A_Ind_ observed by µTR measurement in Figure 3a.

The insets in Figure 4a depict the image and AFM result of one additional ML‐ZrS_3_ nanoribbon with 20 nm thickness and its unpolarized µPL spectrum (with the multiple‐peaks fits) is displayed in the bottom. The unpolarized µTR and micro‐transmittance (i.e., Tr) spectra at 10 K are also included for comparison in Figure 4a. All the excitonic transitions of A_Ind_, A_S1_, A_1_, A_2_, A_S2_, and A_S_ with energy order show approximately a one‐to‐one correspondence of energy position (with experimental error) in the unpolarized µTR, Tr and µPL spectra of Figure 4a to identify the co‐existence of these features. In contrast to the unpolarized µPL spectrum of ML‐ZrS_3_ in Figure 2a (see the microscope image of Figure 1d with different nanoribbon orientation), the A_2_ emission is observed in Figure 4a owing to the A_2_ emission presents in‐plane anisotropy and sensitive to the optical‐axis polarization at 10 K. The center location of the absorption edge in the Tr spectrum of ML‐ZrS_3_ is also matched well with the main band‐edge transition A_1_ detected in the µTR and µPL measurements for verification of the quasi‐direct characteristic of ZrS_3_. To see the temperature‐variation behavior of the A_X_ series excitons, temperature‐dependent µPL and µTR measurements of 10 – 300 K are implemented. Figure 4b,c respectively show the unpolarized µPL spectra (also the 2D contour plot) of ML‐ZrS_3_ at 10–100 K and at 100–300 K. At 10 K, the broadened peak at lower‐energy side consisted of a prominent indirect‐resonant emission D_I_ (green‐area fit) and a smaller indirect bound‐exciton emission A_Ind_ (brown‐area fit). As the temperature increases to 60 K, the PL intensity of D_I_ decreases faster than that of A_Ind_ to make a broadened D_I_+A_Ind_ peak in Figure 4b. The A_Ind_ excitonic feature is maybe ionized at T = 100‐120 K due to its free to bound behavior in Figure 4c and then the combination peak of D+A_Ind_ is present as a shoulder peak at 150 to 180 K in Figure 4c. The D+A_Ind_ peak will finally merge with the main A_1_ peak to render a combined and broadened µPL peak at ≈1.94 eV at 300 K in ML‐ZrS_3_. Figure S6a (Supporting Information) also presents the quasi‐direct band edge of ML‐ZrS_3_ measured by µPL (with area mapping), Tr and µTR is matched at 300 K, and which is positioned at ≈1.94 eV.

For the temperature variation of the A_S1_, A_S2_, and A_S_ series excitons (i.e.*, a*‐polarized), the fitted intensities of PL in Figure 4b at 10 K have the strength order of A_S1_ (the highest, dark‐blue area fit), A_S2_ (the middle, blue‐area fit), and A_S_ (the lowest, yellow‐area fit) such as the excitonic‐series scheme shown in Figure 2b. As the temperature is increased, the PL intensities of the three excitons are simultaneously degraded and their transition energies are decreased in Figure 4b,c (with scale magnification ×10). The fitted results of A_S1_, A_S2_, and A_S_ can be resolved only up to 150–180 K and they will be merged into a broadened peak when T ≥ 210 K in Figure 4c. For the main band‐edge transition of the A_1_ feature, the peak position is at ≈2 eV at 10 K, the prominent peak feature shows intensity degradation and energy reduction behavior as the temperature increases from 10 to 100 K in Figure 4b as well as from 100 to 300 K in Figure 4c. It will finally dominate the PL emission by merging with the other peak (like D_I_ feature) to form a broadened peak at ≈1.94 eV at 300 K. The A_1_ exciton shows a *b*‐polarized behavior as the indication shown in Figure 2d. The temperature‐dependent PL intensity change of the A_1_ and A_S1_ features can be analyzed to obtain the activation energies for exciton emission. The obtained values are 30 ± 2 meV for A_1_ and 18 ± 3 meV for the A_S1_ exciton as shown in the analysis of Figure S6d (Supporting Information). Figure 4d shows the temperature‐dependent unpolarized µTR spectra (with Lorentzian line‐shape fits) in the temperature range between 10 and 300 K for illustration of the temperature variation of the band‐edge transitions. The 2D contour plot of the µTR spectra of ZrS_3_ is also displayed in the inset. Essentially, the excitonic transitions of the A_X_ series observed in ML‐ZrS_3_ (at the same temperature) present comparable transition energies (within a standard error) detected from both µPL and µTR experiments. The A_Ind_ feature of indirect donor‐bound exciton is gradually ionized at 100–120 K in Figure 4d. The ionization temperature is close to the µPL result in Figure 4c. The temperature‐dependent transition energies of some selected features in the A_X_ exciton series from both experiments in ML‐ZrS_3_ are depicted in Figure 4e with representative error bars. The solid (open) symbols with error bars are the data points of A_S1_, A_S2_, A_S_, A_1_, and B transitions obtained by µTR (µPL) and the solid lines are the least‐square fits of a Varshni empirical formula, E(T) = E(0)‐α⋅T^2^/(β+T), where α is the strength of exciton‐phonon coupling and β is related to the Debye temperature of the material. From the Varshni fits, the Debye temperature related parameter is about β = 280 ± 50 K in this material and the value of exciton‐phonon coupling strength is α = (7.76 ± 0.25)×10^−4^ eV K^−1^ for the A_S1_, A_S2_, and A_S_ series. The values of α of the B (≈2.2 × 10^−4^ eV K^−1^) and A_1_ (≈3.2 × 10^−4^ eV K^−1^) transitions are smaller than that of the As series to show a slower temperature‐energy shift as the temperature is changed in Figure 4e. It is inferred that the lattice dilation along Γ‐X (A_S1_, A_S2_, and A_S_) and along Γ‐Y (A_1_ and B) could present different temperature coefficient due to the in‐plane anisotropy of crystal lattice of the ZrS_3_ nanoribbon.

Figure 4f shows the temperature‐dependent TRPL decay curves of the ML‐ZrS_3_ nanoribbon positioned at the main A_1_ peak between 10 and 300 K for characterization of carrier dynamics of the main band‐edge emission. The solid curves represent the experimental analyses of the TRPL spectra using the exponential‐decay fit (i.e., y = y_0_+I_1_⋅*exp*[‐(*t*‐*t_0_*)/τ_1_]+I_2_⋅*exp*[‐(*t*‐*t_0_*)/τ_2_]) with the decay time constants of τ_1_ and τ_2_ starting from the electron‐hole recombination time at *t* = *t_0_*. The decay time τ_1_ is related to the band‐edge emission lifetime and τ_2_ correlates with the surface state or defect related mechanism in ZrS_3_. The TRPL curves in Figure 4f shows the PL decay time of τ_1_ and τ_2_ are increased as the temperature is lowered from 300 down to 10 K due to the enhanced trapping effect from the surface states and defects in ZrS_3_. When the temperature is lowered down the phonon population is lower and the exciton population is higher, hence the probability of non‐radiative recombination is also lower. The excitons can thus survive longer period to emit light with a longer decay lifetime.^[^ ^48^ ^]^ The inset in Figure 4f depicts the values of τ_1_ of the band‐edge emission are increased linearly from 0.31 to 5.5 ns at 300–10 K. The short decay time (fast) of τ_1_ certifies that the A_1_ exciton is a direct‐recombination emission even it is measured inside a “quasi‐direct” 1D nanoribbon of ML‐ZrS_3_. Figure S6a–c (Supporting Information) respectively show the TRPL mapping results of a bulk ZrS_3_ in a 40 × 40 µm^2^ area at 300 K. The averaged emission wavelength of the A_1_ exciton is ≈639 nm (1.94 eV) and it possesses an averaged PL decay time constant of τ_1_ = 0.31 ns for rendering a direct‐like semiconductor behavior with in‐plane optical anisotropy. Thus, the ML‐ZrS_3_ nanoribbon could own a great potential for fabrication of in‐plane anisotropic optoelectronics devices.

**Figure 5:**
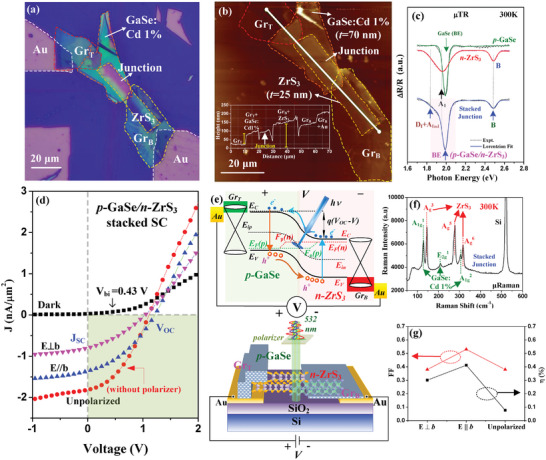
a) The optical microscope (OM) image of a *p*‐GaSe/*n*‐ZrS_3_ stacked heterojunction solar‐cell device. b) The thickness information and AFM image of the *p*‐GaSe/*n*‐ZrS_3_ stacked heterojunction device. c) The µTR spectra of multilayer *p*‐GaSe (green), multilayer *n*‐ZrS_3_ (red) and the stacked junction part (blue). d) The polarized *J*–*V* curves of the *p*‐GaSe/*n*‐ZrS_3_ stacked solar cell under dark, E ⊥ *b*, E || *b*, and unpolarized condition that illuminated by the incident laser of 532 nm between the bias range of −1 to 2 V. e) The band‐edge scheme for operation of the *p*‐GaSe/*n*‐ZrS_3_ homojunction‐like SC under light illumination. The lower part depicts the stacking‐layers structure of the *p*‐GaSe/*n*‐ZrS_3_ homojunction‐like SC. f) The µRaman spectrum in the stacked junction part of the *p*‐GaSe/*n*‐ZrS_3_ SC. g) The result of filling factor (FF) and photoelectric conversion efficiency (η) analyzed from the polarization‐dependent *J*–*V* curves in (d).

**Figure** **5**a shows the microscope image of a stacked multilayer ZrS_3_ and multilayer GaSe doped Cd 1% (i.e., *p*‐type GaSe) heterojunction device with the bottom and top graphene (Gr_B_ and Gr_T_) acted as the ohmic‐contact layer to the Au electrode. The heterojunction device is made on a SiO_2_/Si substrate with all the thickness information of the 2D materials shown in the atomic‐force microscope (AFM) result of Figure 5b. The thickness of the ML‐ZrS_3_ is ≈25 nm and that of the ML *p*‐GaSe is ≈70 nm for making a vertically stacked *p*‐GaSe/*n*‐ZrS_3_ heterojunction‐diode solar cell (SC). The as‐grown layered ZrS_3_ presents lightly *n*‐type conductivity with a carrier concentration of 1.37 × 10^12^ cm^−3^ and electron mobility of 32.11 cm^2^ V‐s^−1^ measured by resistivity and Hall measurement at 300 K. For *p*‐GaSe, the incorporation of 1% Cd in the layered GaSe significantly enhances its *p*‐type behavior and the values of hole concentration and Hall mobility are determined to be 3.4 × 10^16^ cm^−3^ and 19 cm^2^ V‐s^−1^ as listed in Table S2 (Supporting Information). The *p*‐GaSe:Cd 1% is a layered direct semiconductor with a remarkable photoluminescence and high optical absorption, making it a good candidate for solar cell application. GaSe presents an isotropic hexagonal layer structure with a bandgap of ≈1.992 eV at room temperature,^[^ ^49^ ^]^ which aligns well with the quasi‐direct bandgap of *n*‐ZrS_3_ (A_1_ ≈ 1.94 eV) for forming a “homojunction like” *p*‐*n* diode and SC. The *n*‐ZrS_3_ multilayer is stacked along *b* axis and the property of *p*‐GaSe is isotropic (i.e., see the polarized photoconductivity result in Figure S9, Supporting Information) so that the vertically stacked *p*‐GaSe/*n*‐ZrS_3_ “homojunction like” SC is intended to design for polarization sensitive photovoltaic device as shown in Figure 5a,b.

The stacked junction of *p*‐GaSe/*n*‐ZrS_3_ has also been verified by optical measurement of µTR and structural measurement of µRaman. The results are respectively shown in Figure 5c,f for illustration. For *n*‐ZrS_3_ multilayer, the main direct transition is at ≈1.95 eV (red fitted curve) and the main band‐edge transition of *p*‐GaSe [i.e., GaSe (BE)] is at 1.99 eV for rendering a “homojunction like” stacked (hetero‐) junction with an averaged transition energy of BE = 1.982 eV presented in the lower µTR spectrum of the *p*‐GaSe/*n*‐ZrS_3_ stacked junction in Figure 5c at 300 K. The B transition at ≈2.488 eV was simultaneously detected in *n*‐ZrS_3_ part and the *p*‐GaSe/*n*‐ZrS_3_ stacked junction for verification of the formed *p*‐*n* junction. The D_I_+A_Ind_ transition has also be detected as a broadened µTR shoulder feature at ≈1.81–1.85 eV in the *p*‐GaSe/*n*‐ZrS_3_ stacked junction part. For the µRaman measurement of the stacked junction, Figure 5f shows three active modes of A_g_^3^, A_g_^5^, and A_g_^6^ (red color) of ZrS_3_ (referred to Figure 1d) which mixed with three modes of A_1g_^1^, E_2g_^1^, and A_1g_^2^ (green color) originated from layered GaSe^[^ ^49^ ^]^ can be simultaneously detected on the stacked junction of the *p*‐GaSe/*n*‐ZrS_3_ SC. It verifies that the vertically stacked heterojunction of *p*‐GaSe on *n*‐ZrS_3_ is successfully fabricated. Figure 5d shows the current‐density versus bias (*J*–*V*) curves of the multilayer *p*‐GaSe/*n*‐ZrS_3_ heterojunction SC with different illumination conditions of dark, E || *b*, E ⊥ *b* and unpolarized lights with respect to the ML *n*‐ZrS_3_’s longer crystal edge of *b* axis. The incident light source is a 532‐nm solid state laser via the facilitation of light‐guiding microscope (LGM) consisting of an objective lens for impinge onto the junction area of the van der Walls stacked *p*‐GaSe/*n*‐ZrS_3_ SC. The representative scheme of the axially‐polarized photoelectric‐conversion response measurement of the *p*‐GaSe/*n*‐ZrS_3_ stacked junction SC is depicted in the lower part of Figure 5e. The selection of the 532‐nm incident light is referred to that the maximum photoconductivity (PC) response of ZrS_3_ is close to 2.33 eV as shown in Figure S7b (Supporting Information), which covers all photoconductive contributions from the band‐edge transitions (including A_Ind_ and the A_X_ series excitons) as well as close to the exciton B observed in µTR. When the incident photon energy of green laser is larger than those of the indirect (≈1.81–1.85 eV) and direct bandgaps (≈1.94 eV) at room temperature, the energy states of carriers between 1.81 and 2.33 eV of ZrS_3_ will be excited and the energetic carriers of excitation will finally relax their energies (i.e., lost energy to lattice) to the band edge of CBM and VBM for resulting in photoconduction behavior. Because the VBMs and CBMs at different *k* are the main contribution to the photoconduction of the ML‐ZrS_3_, thus the *p*‐GaSe/*n*‐ZrS_3_ stacked SC shows in‐plane anisotropic photoelectric conversion behavior when illuminated with a 532‐nm laser of different polarizations onto the ML‐ZrS_3_. The polarization states of the 532‐nm laser can be controlled by a rotatable visible dichroic‐sheet polarizer and the power densities are measured to be 1 × 10^6^ W m^−2^ for unpolarized condition, 1.88 × 10^5^ W m^−2^ for E || *b*, and 1.01 × 10^5^ W m^−2^ for the E ⊥ *b* situation, respectively.

Figure 5d reveals an increase in the short‐circuit current density (denoted as J_SC_) generated under different illumination conditions of dark, E ⊥ *b*, E || *b*, and unpolarized light from a 532‐nm laser with different power density. The band diagram of the *p*‐GaSe/*n*‐ZrS_3_ “homojunction like” stacked SC under light illumination is depicted in the upper part in Figure 5e. Under dark condition, the built‐in potential (V_bi_) of the SC diode can be measured directly by Kelvin‐probe work function measurement with area mapping for the layered *p*‐GaSe: Cd 1% and *n*‐ZrS_3_ as shown Figure S8a,b (Supporting Information). The averaged work function (Φ) of *n*‐ZrS_3_ is ≈4.965 eV and that of *p*‐GaSe is 5.395 eV to make a value of V_bi_ ≈ 0.43 eV as shown in Figure S7c (Supporting Information) in this homojunction‐like *p*‐*n* diode. Figure S8d (Supporting Information) also reveals a real work function difference for a stacked *p*‐GaSe/*n*‐ZrS_3_ junction device to indicate the contact built‐in potential is ≈0.43 eV. The V_bi_ value is matched with the cut‐in voltage of the *J*–*V* curve under dark condition as shown in Figure 5d. The photoelectric conversion efficiency (η) and filling factor (FF) of the *J*–*V* curves for the *p*‐GaSe/*n*‐ZrS_3_ stacked SC can be calculated from J_SC_ and open‐circuit voltage (*V*_OC_) under different illumination conditions of E ⊥ *b*, E || *b*, and unpolarized light. The filling factor can be evaluated as FF = P_M(ele)_/(*J*_SC_ × *V*_OC_) and the photoelectric conversion efficiency (η) is estimates by η = P_M(ele)_/P_in(opt)_ (×100) %. Where P_M(ele)_ is the maximum electric power (J × V) defined as the maximum rectangular area limited by the fourth quadrant of the *J*–*V* curve under different illuminated condition and P_in(opt)_ represents the incident optical power density.

The estimated values of FF and η calculated from the *J*–*V* curves of different illumination conditions of E ⊥ *b*, E || *b*, and unpolarized light are depicted in Figure 5g and the numbers are listed in Table S3 (Supporting Information) for comparison. Under light illumination, the open‐circuit voltage V_OC_ increases from V_bi_ ≈ 0.43 V (dark) to *V*_OC_ = 1 V owing to the stacked *p*‐GaSe/*n*‐ZrS_3_ SC owns high optical sensitivity to make a larger photocurrent under different illuminated conditions of E ⊥ *b*, E || *b*, and unpolarized light. The FF values of E ⊥ *b*, E || *b* and unpolarized conditions are 0.38, 0.53, and 0.38, and which makes the photoelectric conversion efficiency of the stacked *p*‐GaSe/*n*‐ZrS_3_ SC of *η* = 0.301%, *η* = 0.412%, and *η* = 0.077%, respectively. The maximum η value is along E || *b* polarized direction and the value of *η* = 0.412% is comparable with the other vdW heterostructure of GaSe‐MoSe_2_ with thickness ≈79–118 nm (*η* = 0.46%)^[^ ^50^ ^]^ and a back‐gate controlled *p*‐GaTe/*n*‐MoS_2_ stacked junction on SiO_2_/Si illuminated by a 473‐nm laser (*η* = 0.45%).^[^ ^51^ ^]^ But the efficiency of *η* = 0.412% of the E || *b* condition for the *p*‐GaSe/*n*‐ZrS_3_ SC is lower than a monolayered direct‐gap WSe_2_‐MoS_2_ lateral *p*‐*n* heterojunction solar cell (*η* = 2.56%).^[^ ^52^ ^]^ The relative lower efficiency maybe somewhat relates to the excitonic nature within the vertically stacked ML‐ZrS_3_ interface involving both indirect and quasi‐direct excitons. Additionally, exploring the TMTCs‐based solar cells remains challenging due to limited literature as most assessments are only based on theoretical prediction. For the unpolarized condition, the reduced η value as comparing to those of the E ⊥ *b* and E || *b* polarization is possibly due to the unpolarized optical power is the highest and the filling factor is reduced for decreasing conversion efficiency.^[^ ^53^ ^]^ It is noticed that the FF and η values are the highest under the E || *b* polarized condition for the *p*‐GaSe/*n*‐ZrS_3_ stacked SC. The reason is owing to the indirect gap (D_I_+A_Ind_) and direct main exciton (A_1_) are all *b*‐polarized transitions at θ = 0° observed in µTR and µPL for efficient generation of photocarriers. This situation was also verified by a chain‐like layered ReSe_2_ *p*‐*n* homojunction SC with *b*‐polarized indirect and direct gaps.^[^ ^54^ ^]^ The η value of E ⊥ *b* direction of the *p*‐GaSe/*n*‐ZrS_3_ stacked SC is 0.301% in Figure 5g, which shows 26.8% reduction as comparing to that of the E || *b* condition. The *b*‐polarized photocurrent response of a MoS_2_/ZrS_3_ stacking device had ever been observed to enhance along the ZrS_3_’s crystal‐chain edge of *b* axis.^[^ ^55^ ^]^ The photoelectric conversion along *b* and along *a* axis for the multilayer ZrS_3_‐GaSe vertically stacked SC shows significant in‐plane anisotropic effect mainly contributed from the polarized indirect gap (D_I_+A_Ind_) and polarized direct excitons (A_X_) in the band structure of the quasi‐1D ML‐ZrS_3_. Prior to fabricating *p*‐ and *n*‐ZrS_3_ and their *p*‐*n* homojunction light‐emitting diodes, producing the *p*‐GaSe/*n*‐ZrS_3_ homojunction‐like SC holds the promise to showcase in‐plane anisotropic optoelectronics and energy devices using the quasi‐1D ZrS_3_ nanoribbons. The potential use of the polarized ZrS_3_/GaSe stacking SC device can also be utilized as optical switch or optical digital memory for technological application.

## 3 Conclusion

Axially polarized band‐edge transitions with *b*‐polarized (i.e., indirect gap D_I_+A_Ind_, main exciton A_1_ and spin‐orbital‐splitting B) and *a*‐polarized (A_S1_, A_S2_, A_S_ series, and A_2_ excitons) behaviors are first observed and identified by polarized µTR, µPL, and micro‐transmittance experiments in a quasi‐1D ML‐ZrS_3_ nanoribbon from 10 to 300 K. The high‐quality nanoribbon crystals of ZrS_3_ were grown by chemical vapor transport (CVT) method. XRD and HRTEM measurements confirmed the existence of the monoclinic phase within the crystals and revealed a strong *b*‐axis oriented edge, indicating a highly in‐plane anisotropic structure. This anisotropy is further verified by polarized µRaman measurement. The polarized µPL spectra exhibit D_I_ (including A_Ind_) and A_x_ peaks (including A_1_ and A_S_ series excitons), consistent with those of the polarized µTR spectra and theoretical band‐structure calculations along different *k* directions. The observed evidence suggests an indirect‐gap nature of ZrS_3_ and the origin of the A_X_ excitons is from quasi‐direct excitons along Γ‐X or Γ‐Y orientation. The *b*‐polarized A_1_ exciton reveals the highest PL intensity at 10 K and the exciton recombination lifetime is from 5.5 ns decreases to 0.31 ns from 10 to 300 K, similar to that of a direct‐gap emission in 2D materials. Furthermore, we successfully fabricate an anisotropic multilayer *p*‐GaSe: Cd 1%/*n*‐ZrS_3_ van der Waals stacked solar cell with similar bandgap value. The homojunction‐like *p*‐GaSe/*n*‐ZrS_3_ stacked SC presents the highest photoelectric‐conversion efficiency and the largest filling factor along the E || *b* polarized direction with respect to those detected in the E ⊥ *b* and unpolarized conditions. The primary A_1_ exciton, as predicted by the polarized emission spectra, enhances the performance of η under the E || *b* polarization and also achieves 5.4 times higher efficiency as compared to the unpolarized condition. The quasi‐1D ML‐ZrS_3_ nanoribbon exhibits multiple excitonic levels with polarized optical states, which can potentially provide various quanta for applications in quantum computation and data processing technologies.

## 4 Experimental Section

### Growth of ZrS_3_ Layered Single Crystals

The growth of zirconium trisulfide (ZrS_3_) single crystal was carried out by the chemical vapor transport (CVT) method with iodine trichloride (ICl_3_) as the transport agent. The high purity materials (Zr:99.99% and S:99.99%) with stoichiometric composition (ratio of Zr to S is 1:3 with 10 gram powder in total) were first prepared together with an appropriate amount of ICl_3_ (10 mg cm^−3^) were put into a quartz ampoule (20 cm in length and 3 cm in inner diameter). The quartz ampoule was directly cooled with liquid nitrogen and then sealed in a vacuum environment at ≈10^−6^ Torr. The ampoule was then heated at 760 °C for two days in a three‐zone furnace to synthesize the source material. After that, the furnace temperature was adjusted to create a temperature gradient of 760 °C (source zone) → 680 °C (growth zone) for the crystal growth. The ICl_3_ acted as a transport agent that facilitated the vapor transport of ZrS_3_ from source zone to growth zone, where nucleation and crystal growth may occur at the growth zone. The growth process was lasted for 288 h, yielding large red‐rose colored ZrS_3_ layered crystals with an area size of up to ≈1–2 cm^2^ and a thickness of up to 300 µm. The ZrS_3_ crystals had a layered structure with weak van der Waals interaction between the layers, which enables a mechanical exfoliation of different‐thickness ML‐ZrS_3_ nanoflakes onto a SiO_2_/Si substrate using the scotch tapes of different adhesiveness.

### Micro‐Photoluminescence and Time‐Resolved Photoluminescence Measurements

The optical system of TRPL consisted of a laser scanning confocal spectral microscope (LSCSM) with a 50× objective lens (WD = 8 mm), a Horiba HR‐320 spectrometer, and a TimeHarp 260 data acquisition card (PicoQuant). The excitation source was a 375 nm picosecond pulse laser which was driven by a PDL 800‐D diode‐laser driver. A pair of Galvo mirrors from the LSCSM was used for scanning the mapping area of the 2D layered sample. After the excitation by laser, the photoluminescence signal was first collected by the LSCSM, and then the time‐resolved photoluminescence (TRPL) data were obtained using the time‐correlated single photon counting (TCSPC) technique from the TimeHarp 260 card. The SymPhoTime 64 software was used to process and analyze the TRPL data of the multilayer samples on the SiO_2_/Si substrate. One pair of dichroic sheet polarizers (with a visible‐light‐to‐infrared range) equipped on a rotatable holder was utilized for angle‐dependent polarized measurement.

### Micro‐Thermoreflectance (µTR) Measurements

The multilayered ZrS_3_ exfoliated on a SiO_2_/Si substrate was utilized to be measured using micro‐thermoreflectance (µTR) spectroscopy with a white‐light source derived from a 150‐W Xenon‐arc lamp. The white‐light source was then dispersed by a 0.2‐m PTI monochromator with a 1200‐grooves/mm grating for providing the monochromatic light. Incident and reflected monochromatic lights of the multilayer sample were guided by two silica fibers and a light‐guiding microscope (LGM) consisting of an Olympus 50× objective (WD ≈8 mm). The optical alignment of sample and light path could be adjusted through the CCD camera pairing with the LGM. A 4‐Hz ON/OFF heating current (≈0.4 A) was used to generate the modulated heat to the Au‐coated quartz plate and the sample. An NF 5610B lock‐in amplifier was used to implement phase‐lock detection of the averaged normalized reflectance signal of ΔR/R under heat modulation. A Janis liquid‐helium open‐circled cryostat equipped with a Lakeshore 335 digital thermometer controller facilitated the low‐temperature and temperature‐dependent µTR, µPL, and TRPL measurements.

### Fabrication of p‐GaSe:Cd 1%/n‐ZrS_3_ Stacked Heterojunction Device

Few‐layer and multilayer forms of graphite (multilayered graphenes), *n*‐ZrS_3_, *p*‐GaSe:Cd 1% were mechanically exfoliated using different tapes (white or blue tapes of different stickiness). Polydimethylsiloxane (PDMS) was used as a medium to transfer and stack these multilayered materials onto a SiO_2_/Si substrate with pre‐patterned Au electrodes. Both ML materials and PDMS were aligned in a stacking position precisely using a microscope stage with three axial micromanipulators. The atomic‐force microscope (AFM) was used to measure the thicknesses and profile of the stacked‐junction materials. The graphene was used to make an ohmic contact between the 2D semiconductor and the Au electrode. Keithley 230 programmable voltage source and Keithley 6485 pico‐ampere meter were used to measure the *J*–*V* curves of the stacked heterojunction device. The incident light source of 532‐nm solid‐state laser at unpolarized, E || *b* and E ⊥ *b* conditions were controlled and conducted by an integrated RAMaker polarized µRaman system (with objective) onto the sample. An Ophir optical power meter equipped with a broadband thermal sensor estimates the power density at different polarized conditions. A visible dichroic‐sheet polarizer facilitates the implementation of polarization‐dependent photoelectric‐conversion measurement.

## Conflict of Interest

The authors declare no conflict of interest.

## Author Contributions

C.H.H. conceived the idea and supervised the optical and structural characterization. A.S.R. and C.H,H. grew the sample. C.H.H. is responsible for funding acquisition. A.S.R. and Y.X.L performed the structural, optical and electrical measurements. Y.H.P. did the HRTEM and Kelvin probe work‐function measurement. A.S.R. and C.H.H. calculated and analyzed the theoretical and experimental data. C.H.H. and A.S.R. wrote the manuscript.

## Supporting information

Supporting Information

## Data Availability

The data that support the findings of this study are available from the corresponding author upon reasonable request.
