# Supplementary material for: Axially‐Polarized Excitonic Series and Anisotropic van der Waals Stacked Heterojunction in a Quasi‐1D Layered Transition‐Metal Trichalcogenide
Source: Adv Sci (Weinh). 2024 Aug 5;11(38):2406781. doi: 10.1002/advs.202406781 (PMC11481195; doi:10.1002/advs.202406781)
Supplement: Supplementary file 1 — Supporting Information [file ADVS-11-2406781-s001.pdf]

ADVANCED SCIENCE
Open Access

Supporting Information

for *Adv. Sci.*, DOI 10.1002/advs.202406781

Axially-Polarized Excitonic Series and Anisotropic van der Waals Stacked Heterojunction in a Quasi-1D Layered Transition-Metal Trichalcogenide

*Adzilah Shahna Rosyadi, Ying-Xuan Lin, Yu-Hung Peng and Ching-Hwa Ho**

Supplementary Information of

Axially-Polarized Excitonic Series and Anisotropic van der Waals Stacked Heterojunction in a Quasi-One Dimensional Layered Transition-Metal Trichalcogenide

Adzilah Shahna Rosyadi,[a] Ying-Xuan Lin,[a] Yu-Hung Peng,[a] and Ching-Hwa Ho[a,b,*]

[a] *Graduate Institute of Applied Science and Technology, National Taiwan University of Science and Technology, Taipei 106, Taiwan*
[b] *Taiwan Consortium of Emergent Crystalline Materials (TCECM), National Science and Technology Council, Taipei, 106, Taiwan*

*Corresponding author, E-mail address: chho@mail.ntust.edu.tw

Table of Contents:

1. Angle-dependent polarized μRaman spectra of ML-ZrS_3 at room temperature. 2
2. Polar-plot analysis of each Raman mode in ML-ZrS_3 and its respective attribution. 3
3. EDX and XPS spectroscopy data of bulk ZrS_3. 4
4. Polarized transmittance and absorption spectra from θ=0° (E || *b*) to θ=90° (E ⊥ *b*). 5
5. Low-temperature power dependent PL measurement and analysis 6
6. Theoretical band-structure calculations of multilayer ZrS_3. 7
7. Room temperature μTR, micro-transmittance and TRPL mapping of ZrS_3. 8
8. Temperature-dependent axial resistivity and photoconductivity at 300 K. 9
9. Electrical properties of *n*-ZrS_3 and *p*-GaSe. 10
10. Kelvin probe work-function measurement of *n*-ZrS_3, *p*-GaSe and device. 11
11. The measured solar-cell performance of the *p*-GaSe/ *n*-ZrS_3 stacked junction SC. 12
12. The angle-dependent polarized photocurrent response in layered GaSe 13

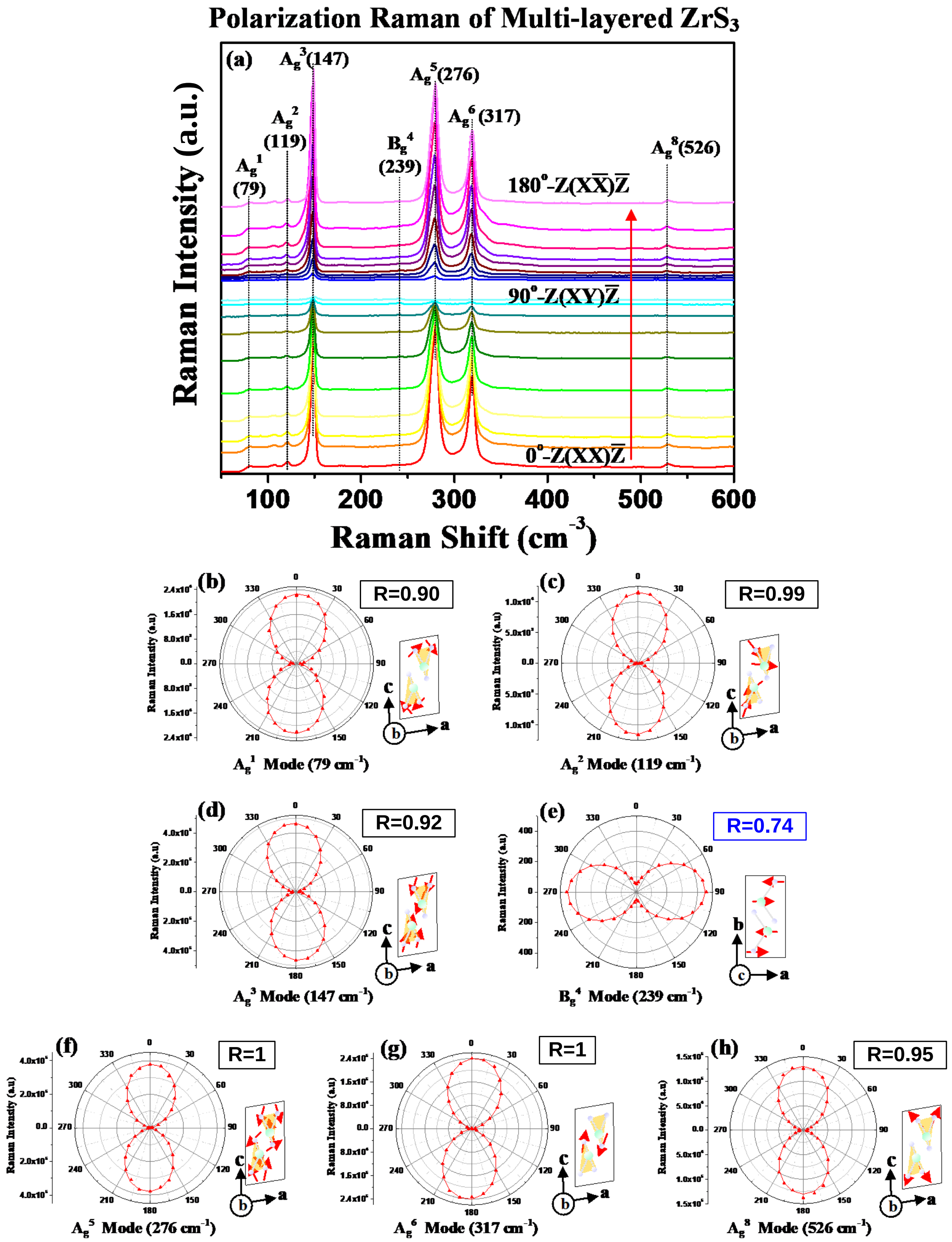

Figure S1. (a) Angle-dependent μRaman measurements of ML-ZrS_3 at room temperature for the polar plots in seven vibrational modes of (b) A_g^1~79 cm^{-1}, (c) A_g^2~119 cm^{-1}, (d) A_g^3~147 cm^{-1}, (e)

B_g^4~239 cm^{-1}, (f) A_g^5~276 cm^{-1}, (g) A_g^6~317 cm^{-1}, (h) A_g^8~526 cm^{-1}. The value of polarized rejection ratio (R) that defined as $R=(I_{max}-I_{min})/(I_{max}+I_{min})$ for the polarized-intensity change of each vibration mode is also shown. The representative schemes of the atomic movement of each mode is also included in the inset in (b) – (h). The most prominent peaks in (a) are the A_g^3, A_g^5 and A_g^6 vibrational modes. These peaks may come from the Zr-S related bonds with intra- or inter-chain vibrations in the ZrS_3 layer. The polar plots of all A_g related modes show a maximum intensity at θ_m=0° and they are fully forbidden at $Z(XY)\bar{Z}$ (θ=90º). The maximum strength of the B_g^4 mode is oriented at θ_m=90° due to its shearing-mode behavior. The A_g-related modes are attributed to the breathing mode. The attributions of the A_g^1 and A_g^8 modes are dominated by the S-S bond movements as shown in Table S1.

Table S1. Fitting Parameters of the polar plots of angular-dependent μRaman results with the equation: $I_\theta = I_o + I_p.cos^2(\theta - \theta_m)$ and their attributions.

Raman Modes	Attribution	I_o (a.u)	I_p (a.u)	θ_m(º)
**A_g^1 (79 cm^{-1})**	S-S layer deformation	3.1	1.45x10^3	0 ± 5
**A_g^2 (119 cm^{-1})**	Zr-S layer deformation	7.3	9.69x10^3	0 ± 5
**A_g^3 (147 cm^{-1})**	Zr-S inter-chain	750	4.18x10^5	0 ± 5
**B_g^4 (239 cm^{-1})**	Zr-S chain shearing	50	1.87x10^2	90 ± 5
**A_g^5 (276 cm^{-1})**	Zr-S intra-chain	2	3.82x10^5	0 ± 5
**A_g^6 (317 cm^{-1})**	Zr-S inter-chain	1.3	2.34x10^5	0 ± 5
**A_g^8 (526 cm^{-1})**	S-S Expansion	146	1.00 x10^4	0 ± 5

(a)

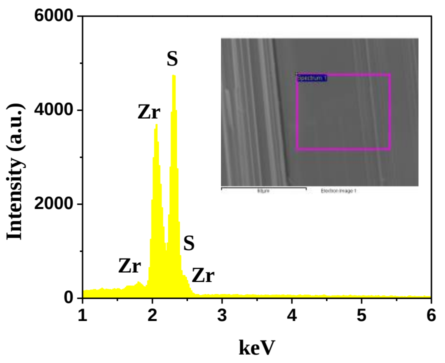

ZrS3	Element	Atomic (%)	Ideal Value (%)
	Zr	25.51	25
	S	74.49	75

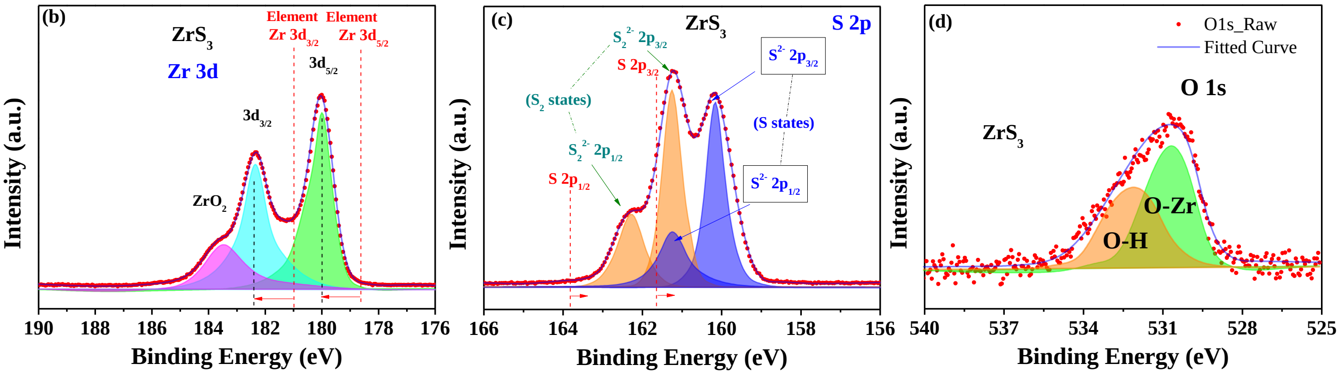

Figure S2. (a) Energy Dispersive X-ray (EDX) Spectroscopy of ML-ZrS_3. The analyzed result of peaks (Zr and S) shows a slight S deficiency in the as-grown layered ZrS_3 crystals. The calculated stoichiometry of Zr and S is listed in the below table. (b) The analysis of X-ray photoelectron spectroscopy (XPS) of Zr 3d orbitals of ZrS_3. The binding energies of Zr $3d_{3/2}$ and Zr $3d_{5/2}$ orbitals will undergo a blue shift from a pure element to ZrS_3 compound. (c) The XPS result of the S 2p orbitals observed in ZrS_3. The peaks include S^{2-} $2p_{3/2}$ (160.2 eV), S^{2-} $2p_{1/2}$ (161.2 eV), S_2^{2-} $2p_{3/2}$ (161.2 eV), and S_2^{2-} $2p_{1/2}$ (162.35 eV) in the ZrS_3 compound. The corresponding energies are lower than those with S $2p_{1/2}$ at 163.8 eV and S $2p_{3/2}$ at 161.7 eV in the pure element. The relative peak intensity of S^{2-} reveals lower than that of S_2^{2-}. (d) The O 1s peak of ZrS_3 to show a little bit oxidation in the crystal.

Polarized transmittance and absorption spectra of ZrS_3

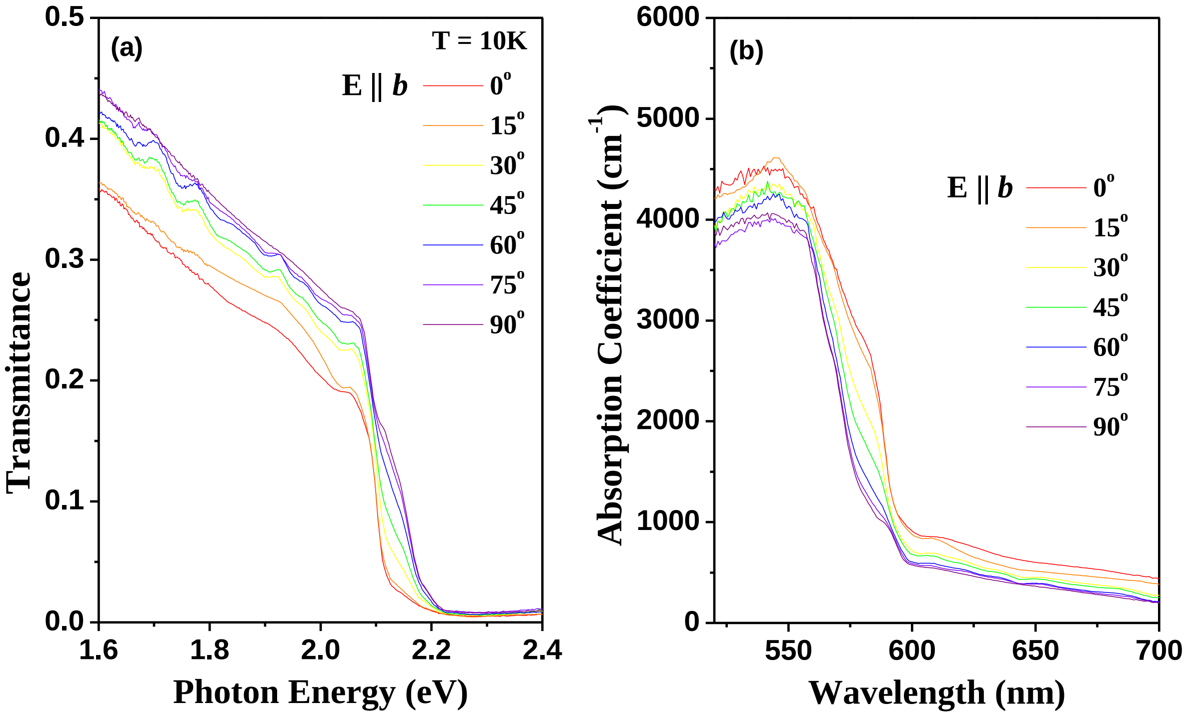

Figure S3. (a) The micro-transmittance spectra and (b) converted optical absorption data of multilayered ZrS_3 with the polarization angle varied from θ=0° (E || *b*) to θ=90° (E ⊥ *b*) near the indirect band edge at 10 K. The E || *b* polarized spectrum dominates the smaller band gap and thus determines the indirect gap of ZrS_3 is along the *b*-polarized direction in the band structure.

Power Dependence of PL Spectra of ML-ZrS_3

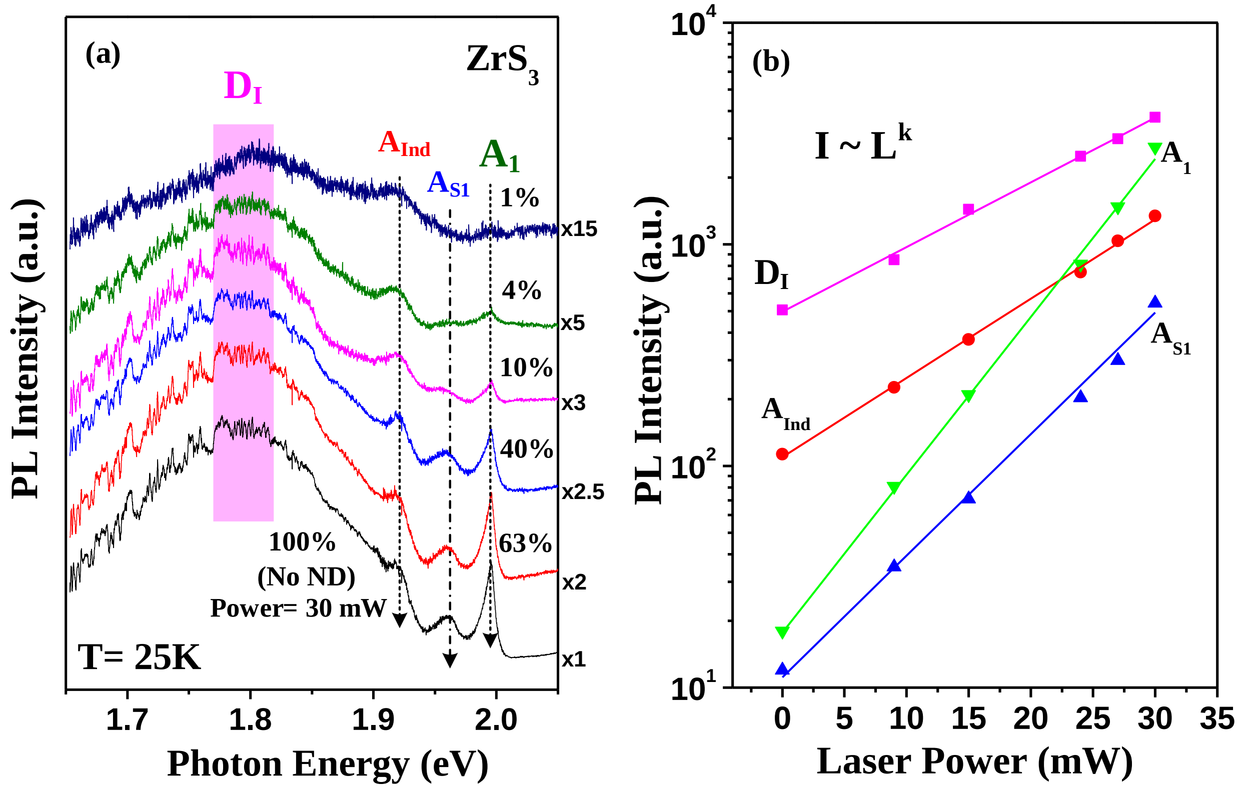

Figure S4. Low-temperature power dependent PL measurement of unpolarized condition for the layered ZrS_3. (a) Power dependent PL spectra of multilayer ZrS_3 (~200 nm thick) at 25 K using a 375 CW laser with different laser power [0.3 mW (1%) to 30 mW (100%) controlled by neutral density filter (ND)] near band edge. Four main PL emissions of D_I, A_{Ind}, A_{S1} and A_1 are observed and their magnification scales (×15 to ×1) are denoted at the right side of each spectrum. (b) The semi-logarithm plot of PL peak intensity versus laser power for each feature of D_I, A_{Ind}, A_{S1} and A_1 derived from (a). The power dependence of each feature (solid line) is analyzed using a law of $I \sim L^k$ [I is the PL intensity and L is the laser excitation power]. The obtained fitted values are k≈1.07 and k≈1.09 for the indirect related parts of D_I and A_{Ind}. For the free-exciton related emissions, the k values are k≈1.18 for A_1 and k≈1.13 for A_{S1}, respectively. The k values of free-exciton emissions are larger than those of the indirect-related emissions in ZrS_3.

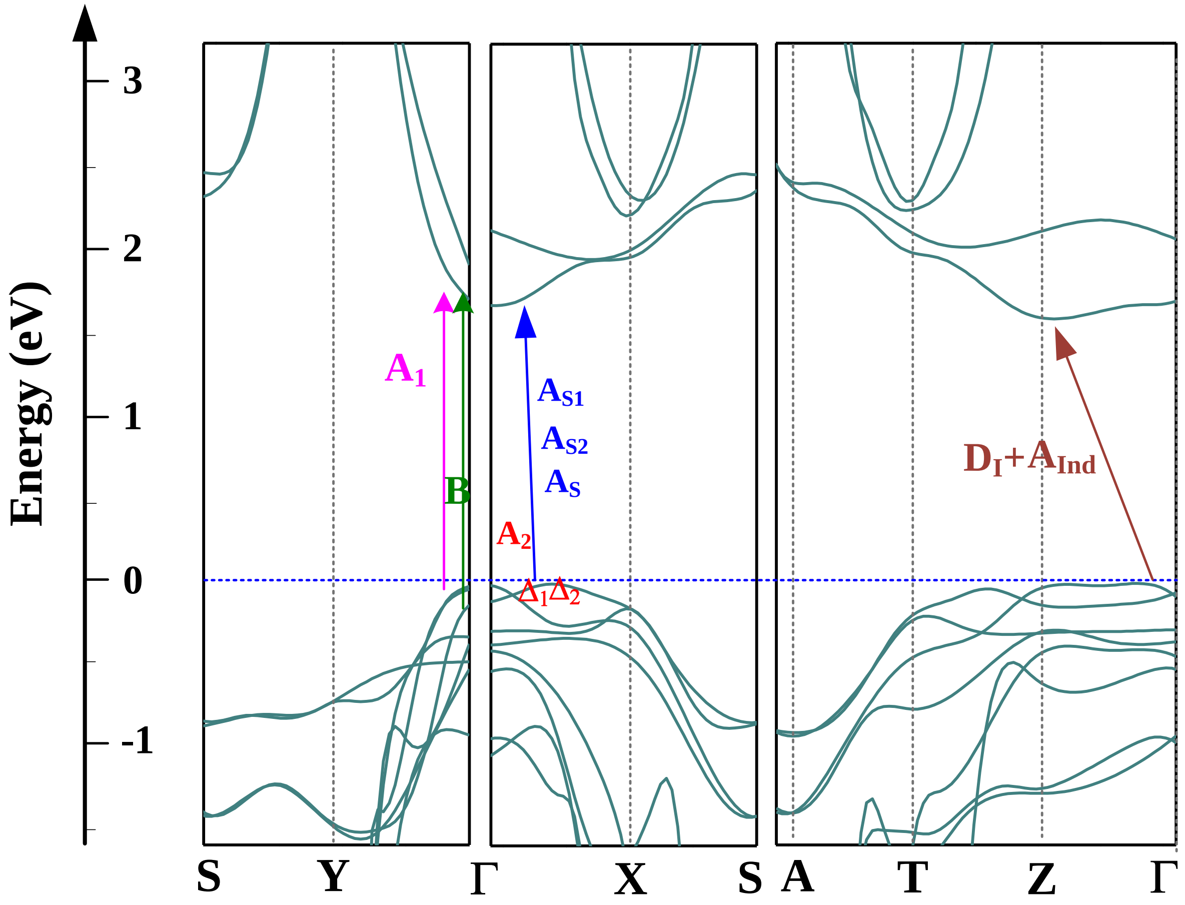

Figure S5. Band-structure calculated result of bulk ZrS_3 using first-principle calculations in the framework of density-function-theory (DFT) basis. The calculation was implemented via the software of VSAP simulation package. Local density approximation (LDA) together with GW and Bethe-Salpeter equation (BSE) were employed for the exchange and correction of potentials. Along with the calculated band structures, the experimental transition features of the D_I+A_{Ind}, A_1, A_2, B, and the A_{S1}, A_{S2}, and A_S series are also assigned and indicated. The CBM is at Z point and the D_I+A_{Ind} features of indirect-like resonant transition and donor-bound exciton are from Γ to Z. The top of valence band is shown to possess multivalley degeneracy like Δ_1, Δ_2, *etc.* along Γ to X and which may also cause A_S series transitions and A_2. The A_1 transition is along Γ to Y and present *b*-polarized behavior and B is from spin orbital splitting (Δ_{SO}) in the ML-ZrS_3 nanoribbon.

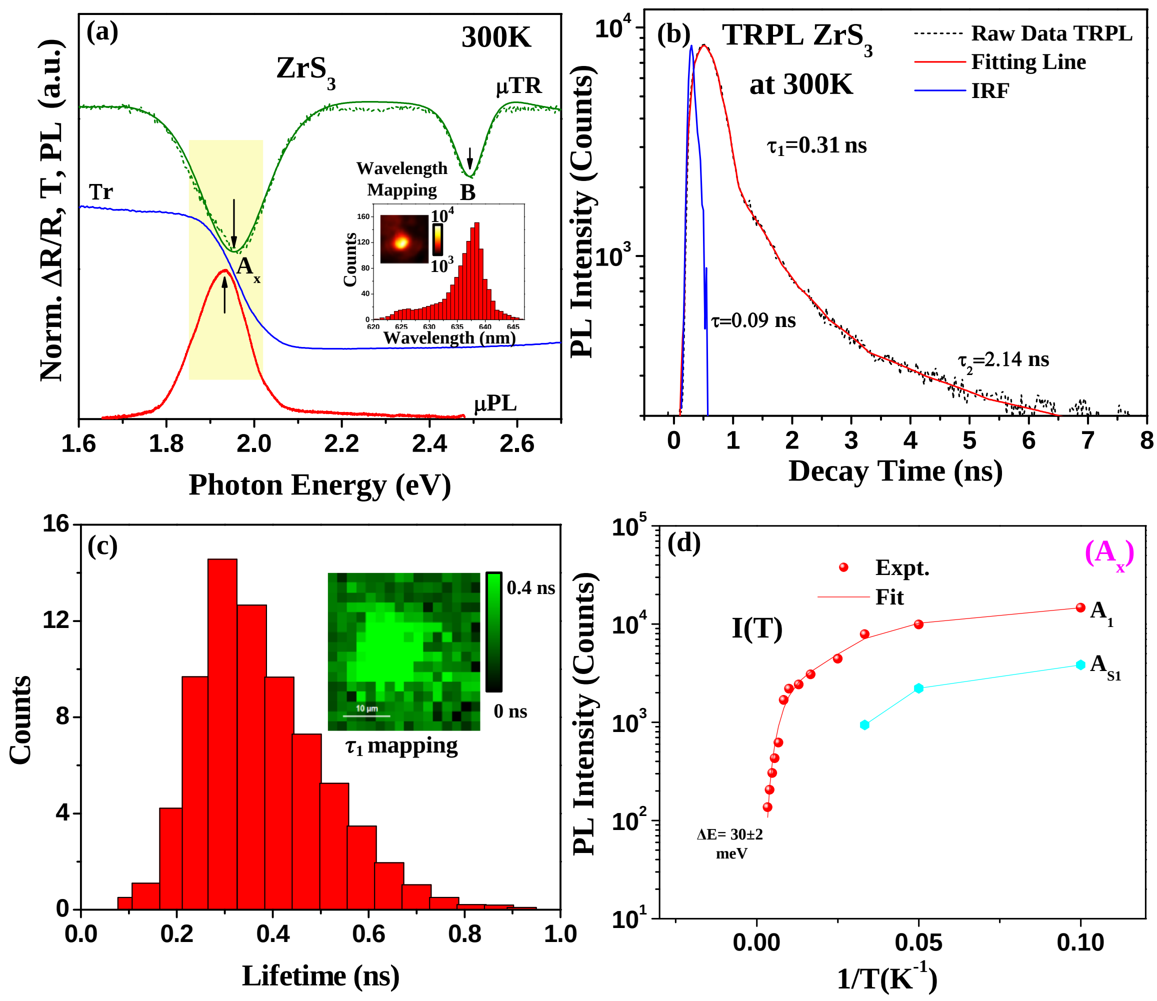

Figure S6. (a) Comparison of quasi-direct band edge in the ML-ZrS_3 at 300 K measured by μTR, micro-transmittance and μPL measurements. The inset shows the area mapping of the PL intensity among 40×40 μm^2 region. The histogram of emission wavelength reveals the main A_1 excitonic emission is at an averaged wavelength of 639 nm (1.94 eV). (b) The averaged TRPL decay curves of the main A_1 peak of ZrS_3 at 300 K. The PL decay fit shows the lifetime of band-edge emission (τ_1) is about 0.31 ns and defect related lifetime (τ_2) is about 2.14 ns. The system IRF response (blue line) indicates a lifetime of 0.09 ns (< 0.1 ns). (c) The fluorescence lifetime-image mapping (FLIM) image and histogram of the band-edge emission lifetime (τ_1) among a 40×40 μm^2 region of bulk ZrS_3. The value is also close to 0.31 ns. (d) The analysis of PL intensity degradation versus temperature change for obtaining the activation energy of the A_1 and A_{S1} excitons in the temperature-dependent μPL spectra of ML-ZrS_3 in Figure 4(b) and 4(c).

Electrical Properties of ZrS_3

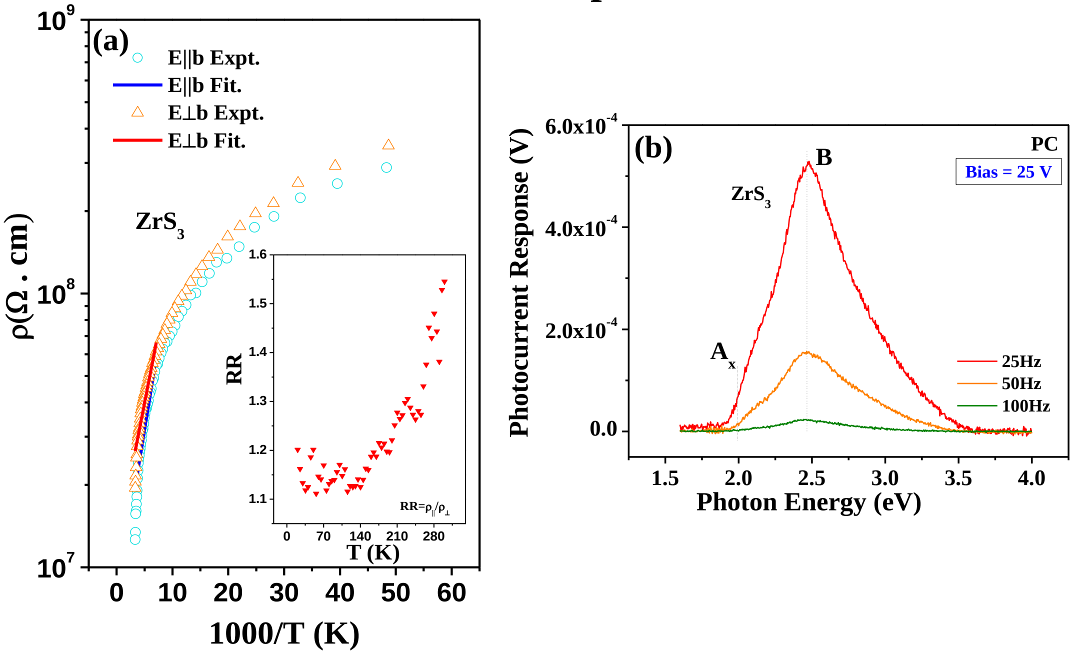

Figure S7. (a) Temperature-dependent resistivity of ZrS_3 in E || *b* and E ⊥ *b* direction. The activation-energy fit is analyzed using the expression $\rho(T)=\rho_0\cdot\exp[\Delta E/(k\cdot T)]$. The inset shows the resistivity ratio (RR) affected by different crystal orientation (*a* to *b* axis) is within 1.1~1.55 (20 to 300 K). (b) Photoconductivity (PC) spectra of ZrS_3 measured at different input frequency of *f*=25, 50, and 100 Hz at 300 K. The maximum peak response is at 2.3-2.4 eV, close to the maximum absorption energy of the 532-nm laser of 2.33 eV. When the incident photon energy of green laser is larger than those of the indirect (~1.81-1.85 eV) and direct bandgaps (~1.94 eV), the energy states of carriers between 1.81 and 2.33 eV of ZrS_3 will be all excited and the energetic carriers of excitation will finally relax their energies (*i.e.* lost energy to lattice) to the band edge of CBM and VBM for resulting in the photoconduction behavior. According to (b), the PC response of band-edge conduction of ZrS_3 will start from its bandgap energy of ~1.805 eV and then increases the response and finally reaches the maximum value of 5.4×10^{-4} (V) from a load resistor at ~2.5 eV. Because the VBMs and CBMs at different *k* are the main contribution to the photoconduction of the ML-ZrS_3, thus the *p*-GaSe/*n*-ZrS_3 stacked SC shows in-plane anisotropic photoelectric conversion behavior when illuminated by a 532-nm laser with different polarizations onto ML-ZrS_3.

Table S2. Electrical Properties of Layered ZrS_3 and GaSe:Cd 1%

**Materials**		**ρ_{300K} (Ω cm)**	**ρ_{20K} (Ω cm)**	**ρ_o (Ω cm)**	**Activation energy ΔE (meV)**	**Hall carrier type**	**Carrier density (cm^{-3})**	**Hall Mobility (cm^2 $V^{-1}s^{-1}$)**
* **ZrS_3**		1.42×10^7				n	1.37×10^{12}	32.11
**GaSe:Cd 1%**		9.66				p	3.40×10^{16}	19.02
** **ZrS_3**	**E ∥ *b***	1.26×10^7	2.88×10^8	3.53×10^5	90.44			
	**E ⊥ *b***	1.95×10^7	3.47×10^8	9.41×10^5	80.69			

(*) obtained by van der Pauw method at 300 K
(**) derived by regular bar-type (four point) resistivity measurement with different orientation.

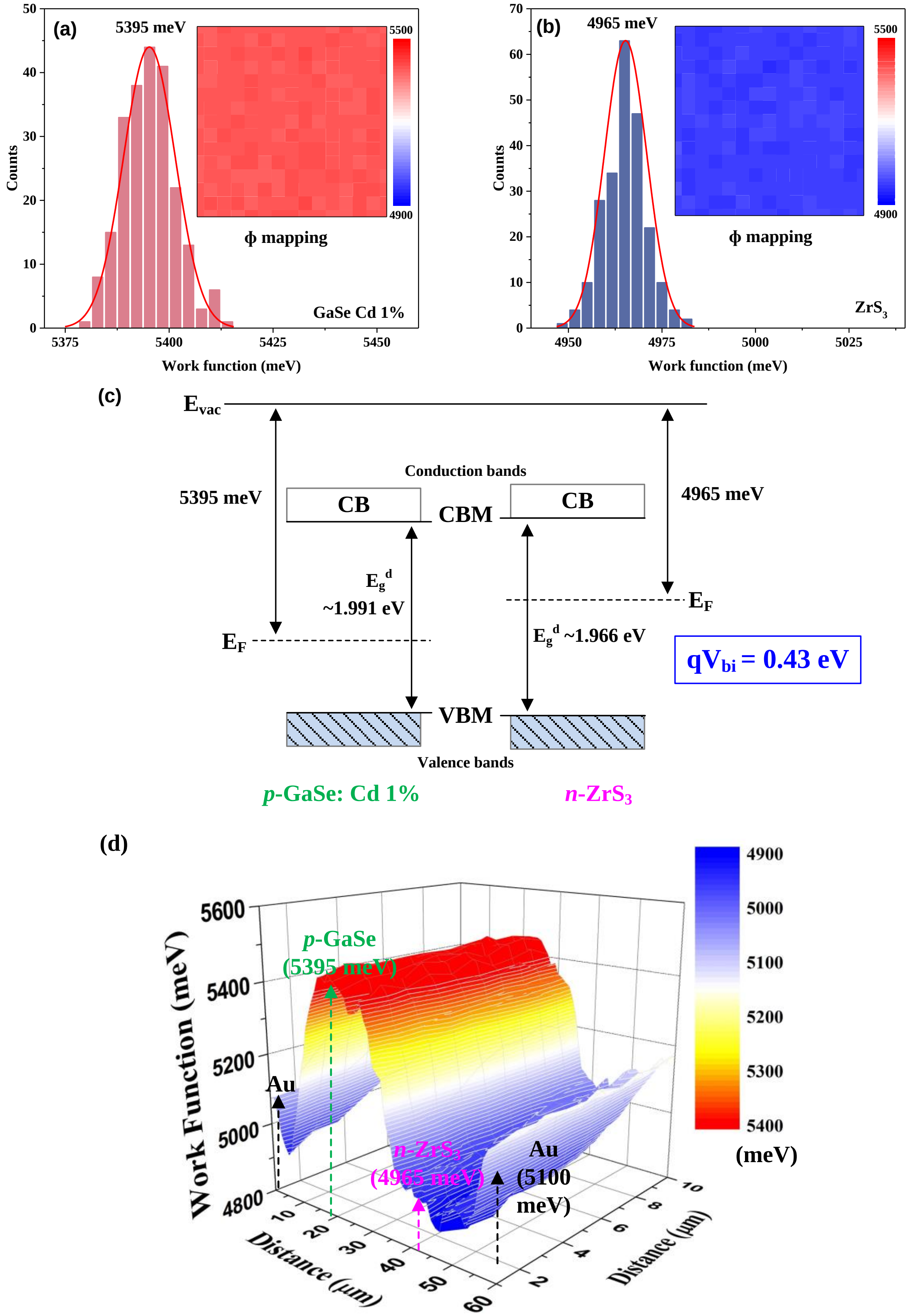

(a)
5395 meV
ϕ mapping
5500
4900
GaSe Cd 1%
Counts
Work function (meV)
(b)
4965 meV
ϕ mapping
5500
4900
ZrS_3
Counts
Work function (meV)
(c)
E_{vac}
5395 meV
4965 meV
Conduction bands
CB
CB
CBM
E_g^d ~1.991 eV
E_g^d ~1.966 eV
E_F
E_F
qV_{bi} = 0.43 eV
VBM
Valence bands
p-GaSe: Cd 1%
n-ZrS_3
(d)
p-GaSe (5395 meV)
Au
n-ZrS_3 (4965 meV)
Au (5100 meV)
Work Function (meV)
Distance (μm)
Distance (μm)
(meV)

Figure S8. Kelvin-probe work function measurement with area-mapping function of layered (a) *p*-GaSe: Cd 1% and (b) *n*-ZrS_3 at room temperature. The averaged work function (Φ) of *n*-ZrS_3 is centered at 4965 meV and that of the *p*-GaSe is centered at 5395 meV. (c) The band scheme includes the information of work function measured by Kelvin probe and the direct gap measured by μTR for both *n*-ZrS_3 and *p*-GaSe. The contact potential of built-in voltage is thus determined to be V_{bi}~0.43 V in the *p*-GaSe/*n*-ZrS_3 stacked heterojunction. (d) The work function profile of a *p*-GaSe/*n*-ZrS_3 stacked heterojunction device measured by Kelvin probe.

Table S3. Estimated solar-cell parameters of the van der Walls stacked *p*-GaSe/*n*-ZrS_3 heterojunction SC from the results of J-V curves in Figure 5(d). Where J_{mp} and V_{mp} identify the maximum rectangular area [*i.e.* the generated electric power $P_{M(ele)}$] of the fourth quadrant of each J-V curve under different illuminated conditions of E ⊥ *b*, E || *b*, and unpolarized light.

**Condition**	**J_{mp}= $P_{M(ele)}/V_{mp}$**	**J_{SC}**	**V_{OC}**	**FF (Fill Factor)**	**Measured Input Laser Power (P_{in})**	***η*** (%)
**E⊥*b***	0.601	0.852	1.000	0.38	1.01×10^5	0.301
**E‖*b***	1.102	1.454	1.000	0.53	1.88×10^5	0.412
**Unpolarized**	1.104	1.962	1.000	0.38	1.00×10^6	0.077
**Unit**	nA/μm^2	nA/μm^2	V	N/A	W/m^2	N/A

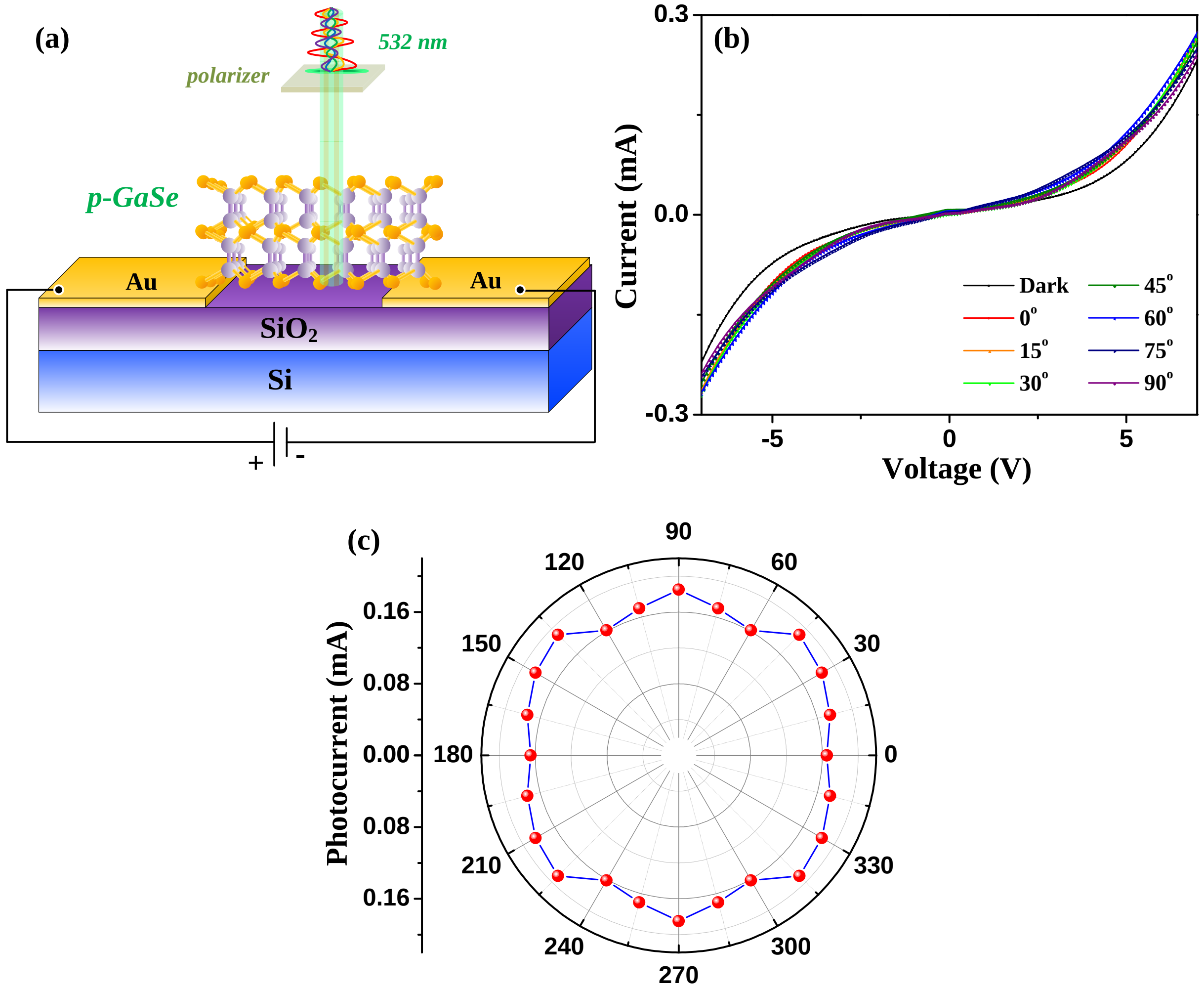

Figure S9. (a) Schematic illustration of photocurrent detection for *p*-GaSe on SiO_2/Si substrate, as observed under the illumination of linearly polarized light with different polarized angle. (b) The V-I characteristics of the *p*-GaSe on SiO_2/Si recorded in ambient dark condition and under varied angles of polarized light (θ=0°, 15°, 30°, 45°, 60°, 75°, 90°). The laser power is ~24 mW. (c) The angle-dependent photocurrent response in layered GaSe measured across a full range of polarization angles (θ=0-360°) to show its isotropic behavior on the van der Waals plane. This observation can further verify the in-plane anisotropic photoconduction behavior of the *n*-ZrS_3/*p*-GaSe heterojunction solar cell may majorly come from the ML-ZrS_3.
